# Splicing of HPV16 E6 promotes aggressive invasion in oropharyngeal cancer via endocytosis of E-cadherin

**DOI:** 10.1101/2025.10.16.682706

**Published:** 2025-10-16

**Authors:** Yvonne X. Lim, Min Liu, Bailey F. Garb, Allison Furgal, Shiting Li, Junsouk Choi, Qingzhi Liu, Huira Kopera, Roland Hilgarth, Marcell Costa de Medeiros, Laura Gonzalez-Maldonado, Thomas Lanigan, Jonathan McHugh, Gregory Wolf, Michelle L. Mierzwa, Veerabhadran Baladandayuthapani, Laura S. Rozek, Maureen A. Sartor, Nisha J. D’Silva

**Affiliations:** 1 Department of Periodontics and Oral Medicine, University of Michigan School of Dentistry, 1011 N. University Ave, Ann Arbor, Michigan, USA; 2 Rogel Cancer Center, University of Michigan, 1500 E Medical Center Dr, Ann Arbor; 3 Department of Biostatistics, University of Michigan School of Public Health, Ann Arbor; 4 Department of Computational Medicine and Bioinformatics, University of Michigan Medical School, Ann Arbor; 5 Department of Human Genetics, University of Michigan Medical School, Ann Arbor; 6 Vector Core, Biomedical Research Core Facilities, University of Michigan Medical School, Ann Arbor; 7 Department of Internal Medicine, University of Michigan Medical School, Ann Arbor; 8 Department of Pathology, University of Michigan Medical School, Ann Arbor; 9 Department of Otolaryngology, University of Michigan Medical School, Ann Arbor; 10 Department of Radiation Oncology, University of Michigan Medical School, Ann Arbor; 11 Department of Oncology, School of Medicine, Georgetown University, Washington D.C.

## Abstract

Human papillomavirus-positive oropharyngeal squamous cell carcinoma (HPV+ OPSCC) is now the leading HPV+ cancer in the United States and United Kingdom. Despite high cure rates, a significant subset of patients have aggressive HPV+ OPSCC that recurs; deciphering the underlying mechanisms will identify biomarkers that delineate patient subgroups for personalized treatment. In a comprehensive investigation using complementary clinically-relevant models of HPV+ OPSCC, we demonstrated that elevated expression of a HPV16 E6 spliced isoform (E6*I) compared to full-length E6 (E6FL), is critical for aggressive invasion. Introduction of splice switching oligonucleotides (SSOs) to inhibit E6FL splicing, effectively mitigated invasion. Mechanistic studies revealed that aggressive invasion occurs via E6*I-induced endocytosis of E-cadherin (ECAD) from the cell membrane, consistent with partial epithelial-to-mesenchymal transition (p-EMT). The clinical relevance of this mechanism was validated in patient cohorts where a reduced ratio of E6FL to all E6 splice variants was associated with high p-EMT signature, lower membrane:cell ECAD, and worse recurrence-free survival. Together, our findings show that low membrane:cell ECAD ratio in pre-treatment biopsies of HPV+ OPSCC, could stratify patients according to risk; patients with a low ratio may not be candidates for treatment de-intensification trials. Importantly, since ECAD detection is by immunohistochemistry, which is widely used even in diagnostic pathology laboratories with limited resources, membrane:cell ECAD ratio could be a clinically scalable biomarker. Targeting E6FL splicing with SSOs should be explored further as a logical strategy for therapeutic intervention in patients with HPV+ OPSCC.

## Introduction

The incidence of human papillomavirus-positive (HPV+) oropharyngeal squamous cell carcinoma (OPSCC) has surged (1–3), overtaking cervical cancer as the most prevalent HPV+ cancer in the United States and United Kingdom (2, 3). The standard treatment protocol is intense chemoradiation, i.e. 70Gy radiation with concurrent cisplatin; while effective, it causes significant adverse effects including dysphagia and xerostomia, which severely diminish quality of life (4–6). Reducing treatment intensity may minimize toxicity, but clinical trials to de-intensify treatment in unselected patient cohorts failed to improve survival outcomes and toxicity profiles (7–10). Furthermore, up to 30% of patients experience tumor recurrence or progression even after standard therapy (11, 12). Even for patients with early stage (I/II) disease, up to 25% may be at risk for distant metastases or loco-regional recurrence after therapy (13–15). These clinical conundrums support that HPV+ OPSCC is a heterogeneous disease and highlight the need for biomarkers beyond clinical staging to stratify patient risk and personalize therapy.

OPSCC is a subset of head and neck squamous cell carcinomas (HNSCC), predominantly driven by HPV16 (16, 17). HPV16 E6 and E7 oncoproteins are the main drivers of OPSCC progression (18). E6 binds ubiquitin ligase E6AP to target the tumor suppressor p53 for degradation (19, 20). E7 targets retinoblastoma (Rb) for degradation (21, 22), which activates transcription factor E2F and its downstream target p16 to promote cell cycle progression (23, 24). Both oncoproteins modulate immune evasion, differentiation, genomic instability, and epithelial to mesenchymal transition (EMT), via p53- and Rb-dependent and -independent mechanisms (25, 26). E6 is expressed as several isoforms, including full-length (E6FL) and spliced variants collectively known as E6* (27). E6*I, resulting from exclusion of 183 bp from E6FL, is the most abundant E6 variant in OPSCC (28–30). While E6FL inhibits p53, E6*I, which is only found in high risk HPV types, does not (27). The functional roles of E6*I and E6FL are poorly elucidated, with a limited number of studies, mostly performed in models of cervical cancer (27). However, due to significant differences in clinical progression, tumor microenvironment, HPV genotype distribution, HPV gene expression and clinical presentation between OPSCC and cervical cancer (7, 31), findings in cervical cancer should not be extrapolated to OPSCC.

In OPSCC, the role of E6*I is controversial. Using bioinformatics approaches, we observed that a shift from E6FL to E6* correlates with poorer survival in 68 human HPV+ HNSCC (28, 32), suggesting that E6* enhances OPSCC progression. However, another study showed that overexpression of E6*I in HPV-negative [HPV(−)] OPSCC cells, enhances radiation-induced cell death and p53 activity (33). In a third study, E6FL, but not E6* or overall E6, was associated with improved survival and reduced oxidative phosphorylation in multiple HPV+ OPSCC cell lines and patient cohorts (34). Consistent with this, our gene expression analysis in HPV+ human HNSCC (majority OPSCC) suggested that higher E6* activity correlates with increased oxidative phosphorylation, keratinization, and DNA damage response (32). These findings in a limited number of studies suggest distinct roles for E6* and E6FL in OPSCC but their contributions to aggressive phenotypes in OPSCC are unclear. Therefore, investigations in appropriate models that recapitulate the biology of OPSCC are needed.

Epithelial-mesenchymal transition (EMT), a driver of invasion and cancer progression, was traditionally viewed as a binary process where cancer cells lost cell–cell adhesion and apical–basal polarity while acquiring mesenchymal characteristics (35). During EMT, loss of epithelial and gain of mesenchymal markers are observed in cancer cells (35). Recent studies propose EMT as a continuum including intermediate states known as partial EMT (p-EMT), where tumor cells co-express epithelial and mesenchymal markers, but may re-localize epithelial proteins from cell-cell junctions to cytoplasm to retain their tumor-initiating capacities while increasing motility (36–38). p-EMT promotes collective migration and is linked to nodal metastasis, perineural invasion, and higher overall grade in oral cavity SCC (38, 39). In other cancers, such as breast, ovarian and pancreatic cancers, p-EMT correlated with poorer prognosis and more invasive tumors (36, 40–43). Although OPSCC exhibits high intratumoral heterogeneity in p-EMT, the prognostic value is unclear (44).

In this study, we explored the impact of splicing of E6FL to E6*I on OPSCC progression. Using multiple clinically-relevant models, we demonstrated that E6*I drives aggressive invasion via p-EMT, characterized by endocytosis of junctional E-cadherin (ECAD). The impact of splicing of E6FL to E6*I on p-EMT and clinical outcomes was validated in patients using RNA sequencing and multiplexed immunofluorescence. Finally, we designed antisense splice-switching oligonucleotides (SSOs) to inhibit splicing of E6FL to E6*I. Overall, our findings identify splicing of E6FL to E6*I as a critical mechanism of OPSCC progression via endocytosis of E-cadherin (ECAD) and p-EMT. The ratio of membrane to cell ECAD could serve as a biomarker to facilitate patient stratification for personalized therapy, and SSOs prevent splicing of E6FL to E6* thereby uncovering a potentially novel treatment.

## Results

### 1. HPV+ OPSCC cell lines express varying proportions of E6FL and E6*I.

**Fig. 1:**
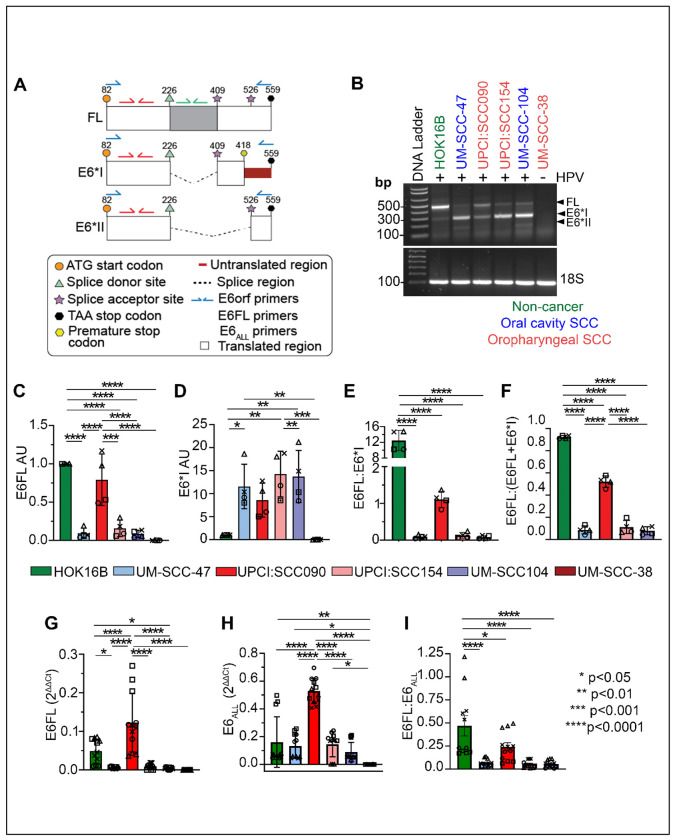
E6*I and E6FL isoforms are differentially expressed in HPV+ cell lines. (A) Schematic indicating primers used to detect E6FL, E6*I and E6*II. (B) Agarose gel detecting E6 isoforms in cDNA from a panel of oral cavity SCC, oropharyngeal SCC, and non-cancer (keratinocyte) cell lines, using primers against E6 open reading frame (E6orf). Expected sizes for E6FL, E6*I and E6*II are 477 bp, 295 bp and 178 bp respectively. (C-F) Quantification of four experiments (including (B)) by densitometric analysis of E6FL and E6*I normalized to 18S (AU = arbitrary units). Each shape shows an independent experiment. (G-I) RT-qPCR of E6FL and all E6 isoforms (E6_ALL_) normalized to β actin. Each shape represents an independent experiment; three points of the same shape indicate three replicates. For all graphs, data are represented as mean ± SD. *p<0.05, **p<0.01, ***p<0.001, ****p<0.0001 (one-way ANOVA post-hoc Tukey test)

In functional and mechanistic studies, we focused on HPV16 because it is the main HPV genotype in over 80% of HPV+ OPSCC (16). The HPV16 E6 gene encodes full-length E6 (E6FL) transcript that can be spliced into shorter isoforms, E6*I and E6*II, collectively known as E6* (27, 30, 45) (Fig. 1A). Recent studies in human HPV+ HNSCC have shown that E6*I is the most abundant among all E6 isoforms, followed by E6FL and E6*II (28, 29, 46, 47). Since expression levels of E6* and E6FL have not been characterized in HPV16+ HNSCC cell lines, we analyzed expression in multiple HPV+ OPSCC and oral cavity squamous cell carcinoma (OSCC) cell lines using complementary approaches. In the first approach, to amplify all E6 variants, primers were designed to span the open reading frame of the E6 (E6orf) gene (Fig. 1A; blue primers). The E6orf forward primer binds from nucleotide 82 (start codon) to 101 and the reverse primer binds from nucleotide 536 to 559. Following PCR amplification, agarose gel electrophoresis was performed to separate E6FL, E6*I and E6*II variants by molecular mass (Fig.1B). We detected two main bands representing E6FL and E6*I (Fig. 1B) with an almost undetectable band corresponding to E6*II (splice donor at position 226 and splice acceptor at position 526) only in HPV+ OSCC cell lines, UM-SCC-47 and UM-SCC-104, but not in HPV+ OPSCC cell lines, UPCI:SCC090 and UPCI:SCC154 (Fig. 1B). This is consistent with previous studies reporting that expression of E6*II is minimal in human HPV+ HNSCC (29, 46). We observed that HPV+ cancer cell lines had lower E6FL expression and higher E6*I expression than HOK16B, a HPV-immortalized non-cancer keratinocyte cell line (Fig. 1C, 1D). Keratinocytes are the cell of origin of OPSCC and OSCC. As expected, the HPV(−) OPSCC cell line, UM-SCC-38, did not express any E6 isoform, and was used as a negative control (Fig. 1B–1D).

To measure the amount of splicing, we calculated: (i) ratio of unspliced E6FL to spliced E6*I (Fig. 1E); and (ii) ratio of E6FL to the total of the two main isoforms, E6FL and E6*I (Fig. 1F). E6*II was excluded due to low detection. Low E6FL:E6*I or E6FL:(E6FL+E6*I) ratios represent increased splicing of E6FL into E6*I. HPV+ OPSCC and OSCC cell lines have lower E6FL:E6*I or E6FL:(E6FL+E6*I) ratios compared to HOK16B, the HPV+ non cancer keratinocytes (48) (Fig. 1E,1F).

The above RT-PCR results were validated in a complementary approach using two additional sets of primers; one (E6FL primer) amplifies the intron region and only recognizes E6FL (Fig. 1A; green primers), while the other (E6_ALL_) amplifies the first exon before the splice region and recognizes all E6 isoforms, including E6FL and E6* (Fig. 1A; red primers). Consistent with initial findings, HPV+ HNSCC cell lines express varying proportions of E6FL and E6_ALL_; with significant variation in the E6FL:E6_ALL_ ratio (Fig. 1G–1H). Notably, the two HPV+ OPSCC cell lines show different splicing profiles, with UPCI:SCC154 having significantly lower E6FL mRNA expression and lower E6FL:E6_ALL_ ratio than UPCI:SCC090 (Fig. 1E, 1F, 1I). As expected, UM-SCC-38, the HPV(−) cell line, does not express either isoform.

Together our findings indicate that E6*I expression is higher in HPV+ cancer cell lines than HOK16B, HPV-immortalized non cancer cells. Also, HPV+ cell lines express varying proportions of E6FL and E6*I transcripts, and E6*II expression is minimal.

### 2. Generation of OPSCC cell lines with stable overexpression of HPV16 E6 isoforms

**Fig. 2:**
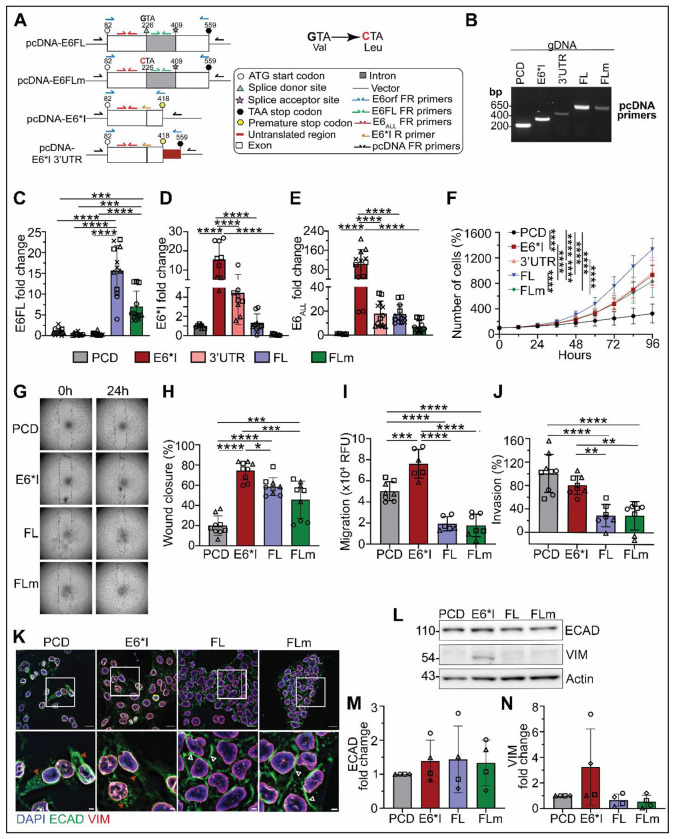
HPV16 E6*I promotes migration and invasion, while HPV16 E6FL promotes proliferation in OPSCC UM-SCC-38 cells. (A) Schematic of E6 constructs and primer design. (B) Agarose gel showing overexpression of E6 isoforms in genomic DNA with PCR using primers against pcDNA vector. Expected size: PCD= 198 bp; E6*I=356 bp; E6*I 3’UTR (3’UTR)=497 bp; FL/FLm=679 bp) (C-E) RT-qPCR of cell lines using primers against full-length E6 (E6FL) (C), E6*I (D) and all E6 isoforms (E6_ALL_) (E). Each shape represents an independent experiment, with three replicates per experiment. Transcript expression was represented as fold change relative to PCD control. (F) Proliferation assay. Viable cells were quantified and expressed as percent (%) of 0h. (G) Representative images of wound healing assay (three independent experiments). (H) Wound closure was quantified from (G) and represents percent (%) closure after 24h. 3 independent experiments with three replicates per experiment. (I&J) Fluoroblok transwell migration (I) and invasion (J) assays. Migration was calculated by normalizing with relative fluorescence unit (RFU) at 0h. Invasion was calculated as a percent of migrated cells. 3 independent experiments were performed, with each shape representing one experiment and each point represents one replicate. (K) Immunofluorescence of DAPI (blue), E cadherin (ECAD, green) and vimentin (VIM, red). Lower panel shows enlarged images from boxed area. Scale bar=100uM (top) and 20uM (lower). Individual channels are in Fig. S2. Brown arrowheads show examples of ECAD in the cytoplasm; white-outlined arrowheads show examples of ECAD at the membrane. (L-N) Representative Immunoblot of ECAD and VIM (L). Actin was a loading control. Densitometry for ECAD (M) and VIM (N) normalized to actin and expressed relative to PCD control. Each data point is from an independent experiment. For all graphs, data are represented as mean ± SD. *p<0.05, **p<0.01, ***p<0.001, ****p<0.0001 (one-way ANOVA post-hoc Tukey test)

Since spliced E6*I is the most abundant E6 isoform in HPV+ cell lines (Fig. 1B), and in HPV+ OPSCC from patients (28, 29, 46), we investigated whether the two main E6 isoforms, E6FL and E6*I, differentially contribute to oncogenic phenotypes in OPSCC. We generated several constructs to express HPV16 E6*I or E6FL. Since splicing of E6FL to E6*I results in a premature stop codon at nucleotide 418 (Fig. 1A), we generated two constructs for E6*I; one with the premature stop codon (termed E6*I) and the other with the original stop codon that comprises the 3’ untranslated region (UTR) from nucleotides 419 to 559 (termed E6*I 3’UTR) (Fig. 2A). To render E6FL non-spliceable while retaining its function (29), we generated E6FLm by mutating G to C at nucleotide 226 (Fig. 2A), which introduces an amino acid change (V42L) at the splice donor site. The nucleotide mutation was verified by sequencing prior to and after transfection. These constructs were stably transfected in UM-SCC-38, an HPV(−) OPSCC cell line that does not endogenously express any E6 isoform. Stable transfection was confirmed in genomic DNA using primers against the plasmid vector (pcDNA) (Fig. 2B), which allows detection of all transfected constructs, including the vector, based on molecular size (Fig. 2B). To verify expression of E6 isoform transcripts, we utilized E6FL and E6_ALL_ primers that detect only E6FL or all E6 isoforms, respectively (Fig. 2A; green and red primers, respectively). We also utilized primers specific to E6*I, i.e., E6*I reverse primer that binds to the exon-exon region to detect E6*I and cannot detect E6FL (Fig. 2A; orange primer). As expected, cells overexpressing E6*I and E6*I 3’UTR showed transcripts with E6*I and E6_ALL_ primers, but none with E6FL primers (Fig. 2C–2E). In contrast, transcripts in cells overexpressing E6FL and E6FLm were detected with both E6_ALL_ and E6FL primers (Fig. 2C, 2E). There was a low amount of E6*I transcript in cells overexpressing E6FL, but not E6FLm (Fig. 2D), showing that cells overexpressing E6FL had partial splicing, but none in E6FLm. To validate these findings, we performed PCR using E6orf primers (Fig. 2A; blue primers), to detect the open reading frame of the *E6* gene in cDNA and the specific E6 isoform was confirmed by molecular size using agarose gel electrophoresis (Fig. S1A). As expected, cells overexpressing non-spliceable E6FLm did not express any E6*I, while cells overexpressing wild-type E6FL expressed E6FL and a small fraction of spliced E6*I (Fig. S1A). Since the E6orf reverse primer is located at the 3’UTR, a signal is observed in cells overexpressing E6*I 3’UTR but not E6*I that lacks the 3’UTR (Fig. 2A; S1A).

Although stably transfected cells expressed transcripts, it is unclear if these cells also express E6 protein. Therefore, we attempted immunoblot analysis on lysates from HPV+ cell lines using two commercially available E6 antibodies; lysates from HPV(−) cell lines were used as negative control (Fig. S1B, S1C). Neither E6 antibody revealed bands corresponding to the expected size of E6FL or E6*I (Fig. S1B, S1C). Since E6FL, but not E6*I, degrades p53 (49–51), we interrogated p53 protein expression in stably transfected cells as a surrogate marker for E6FL and E6*I activity. Overexpression of E6FL and E6FLm suppressed p53 in UM-SCC-38 compared to E6*I (Fig. S1D, S1E). In contrast, both E6*I and E6*I 3’UTR had similar p53 expression as the empty vector (PCD) control (Fig. S1D, S1E). This shows that E6FL and E6FLm, but not spliced E6*I isoforms, downregulate p53 protein expression in OPSCC, similar to findings in cervical cancer. Together these findings verify that the stably transfected cell lines express corresponding E6 isoforms.

### 3. E6*I induces invasion and partial EMT *in vitro*.

Since the association of E6*I with aggressive OPSCC is controversial (28, 32–34), we investigated whether splicing of E6FL to E6*I induces proliferation and spread. All E6 isoforms induced proliferation in UM-SCC-38 compared to control (PCD) (Fig. 2F). Moreover, UM-SCC-38 overexpressing E6FL (expresses both E6FL and E6*I) demonstrated higher proliferation than E6FLm (non-spliceable; does not express E6*I), E6*I 3’UTR, and E6*I (Fig. 2F), suggesting that co-expression of E6FL and E6*I enhances proliferation rather than either alone. Since overexpression of E6*I or E6*I 3’UTR yielded similar proliferation (Fig. 2F) and E6*I expression is higher than E6*I 3’UTR (Fig. 2D), E6*I and not E6*I 3’UTR was used for subsequent experiments.

Locally advanced OPSCC with extranodal extension has been associated with inferior outcomes (13, 15, 52, 53). Since invasion is associated with aggressive spread of HNSCC (54, 55), we performed migration (no extracellular matrix) and invasion (with matrigel) assays. UM-SCC-38 overexpressing E6*I exhibited greater migration (Fig. 2G–2I) and invasion (Fig. 2J) than cells overexpressing E6FL or E6FLm. Although UM-SCC-38 overexpressing E6*I had greater migration than control (PCD) cells (Fig. 2G–2I), invasion did not differ significantly (Fig. 2J). This suggests that E6*I behaves more aggressively than E6FL and E6FLm, and more similarly to HPV(−) HNSCC (represented by UM-SCC-38 with empty vector control), which is aggressive. In later studies, we verified the impact of E6*I on aggressive invasion in multiple HPV+ OPSCC models.

During invasion, SCC may undergo EMT, changing from non-motile epithelial to spindled, motile mesenchymal phenotypes (54, 55). Cancer cells undergoing complete EMT acquire mesenchymal markers such as vimentin (VIM), and lose epithelial markers such as E-cadherin (ECAD) (35), whereas cancer cells undergoing partial EMT (p-EMT) co-express epithelial and mesenchymal markers (38, 56), but may re-localize epithelial proteins from cell-cell junctions to cytoplasm to reduce cell-cell adhesion and increase motility (36, 37). Since E6FL and E6FLm decreased invasion of UM-SCC-38 relative to spliced E6*I (Fig. 2G–2J), we investigated the impact of the E6 isoforms on expression of EMT markers by immunofluorescence. As expected, ECAD, which facilitates cell-cell adhesion (57, 58), was mostly localized at cell junctions in UM-SCC-38 overexpressing E6FL or E6FLm; however in E6*I or control (PCD) cells, ECAD mainly localized in the perinuclear/cytoplasmic region (Fig. 2K; S2), consistent with a p-EMT phenotype (36, 37). In contrast, no change of subcellular localization of VIM was observed (Fig. 2K; S2). Control- and E6*I-overexpressing cells were more isolated and with a spindled morphology compared to FL- and FLm-overexpressing cells that were more tightly packed and polyhedral (Fig. 2K). In a complementary approach, immunoblot analysis revealed that cell lines had similar expression of ECAD, whereas VIM was elevated in E6*I-overexpressing cells, although the increase was not significant (Fig. 2L–2N). These studies support that E6*I potentiates invasion in OPSCC by inducing p-EMT, i.e., re-localization of ECAD from cell-cell junctions to the intracellular region (36–38). In contrast, E6FL (that allows partial splicing to E6*I) promotes proliferation, which suggests differential roles of HPV16 E6 isoforms in OPSCC.

### 4. E6*I enhances aggressive tumor spread *in vivo*

**Fig. 3:**
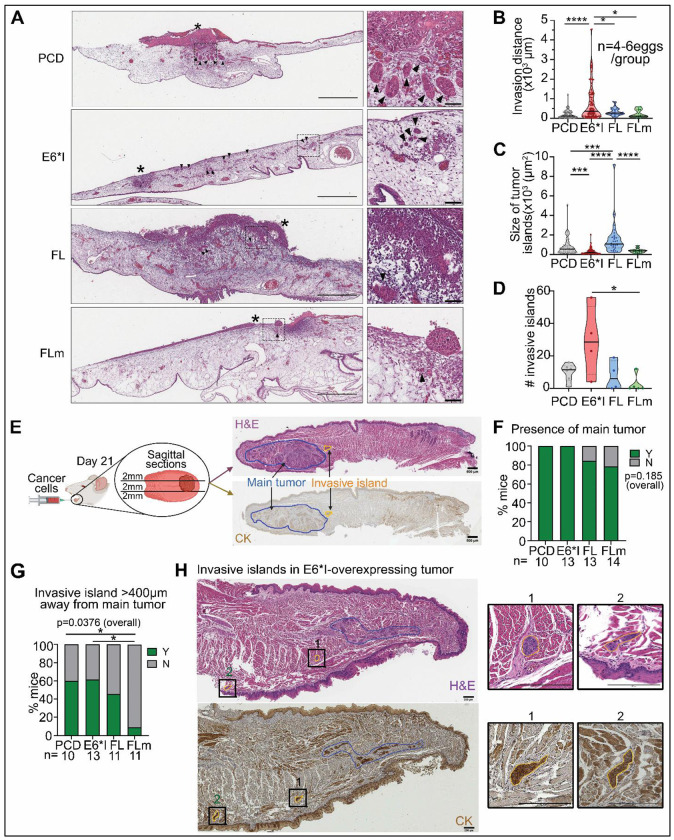
E6*I promotes invasion in vivo. (A) UM-SCC-38 labeled with CellTracker^™^ Green CMFDA Dye were seeded on the upper CAM that was harvested 3 days later, sectioned and stained with hematoxylin and eosin (H&E). Whole-slide imaging of CAM sections was performed. Invasive tumor islands are indicated by black arrowheads. Tumor bulk is indicated by an asterisk. Right panel shows enlarged images from boxed areas in left panels. Scale bar= 500μm (left), 50μm (right). (B-D) Nearest distance of invasive islands from the tumor bulk (B), size of invasive islands (C) and number of invasive islands (D) were quantified from the entire CAM section shown in (A). N=4-6 eggs/group. Each point in (B) and (C) represents an invasive tumor island; each point in (D) represents a different egg. Data are represented as mean ± SD. *p<0.05, ***p<0.001, ****p<0.0001 (One-way ANOVA post-hoc Tukey test). (E) Schematic diagram of mouse experiment. Sagittal sections of tongues were harvested 21 days after tumor injection in mice and stained with H&E and cytokeratin (CK) to highlight epithelial cells. (F) Percentage (%) of mice showing tumor. (G) Percentage of mouse tumors with invasive islands located >400μm from main tumor. *p<0.05 (Fisher’s Exact test). Only statistically significant group comparisons are shown as asterisks; the rest are not significant.(H) Mouse tumor stained with H&E and CK to show invasive islands that are found far away from the main tumor. Blue outline shows main tumor and yellow outline shows invasive island. Box 1 and 2 showed enlarged images of the corresponding invasive islands.

Locoregional spread of OPSCC into adjacent tissues and lymph nodes occurs in aggressive disease and correlates with worse overall and disease-specific survival (13, 52, 59, 60). Since E6*I enhances migration and invasion in UM-SCC-38 *in vitro*, we investigated whether E6*I promotes spread *in vivo* using two models, mouse and previously established CAM models (61). UM-SCC-38 stably expressing E6 isoforms or control (PCD) were grafted on the upper CAM, which was harvested three days later. Whole-slide imaging of hematoxylin and eosin (H&E)-stained sections was performed to visualize predominant patterns of invasion (Fig. 3A). In E6*I-overexpressing OPSCC, tumor islands moved further from the main tumor (Fig. 3B) and were significantly smaller than in E6FL-overexpressing and control groups (Fig. 3C). This is consistent with an aggressive pattern of invasion, where small clusters of tumor cells invade the surrounding tissues (62, 63). Also, E6*I-overexpressing tumors had significantly more invasive islands than E6FLm-overexpressing tumors (Fig. 3D). In the CAM model, during invasion, the basement membrane is altered and degraded to facilitate tumor spread (64). We thus assessed degradation of the basement membrane by staining CAM sections for laminin (Fig. S3A ) (64). E6FLm-overexpressing tumors had a trend towards least disruption of laminin-positive basement membrane (Fig. S3B).

CAM findings were corroborated in athymic nude mice, an independent in vivo model. UM-SCC-38 overexpressing E6 isoforms or control (PCD) were injected into the tongue, which was harvested 21 days later. Tongues were sectioned and stained with H&E and cytokeratin (highlights epithelial cells) to assess invasion (Fig. 3E). Tumors were observed in all mice injected with UM-SCC-38 overexpressing E6*I or control (PCD) cells, and in 80% of mice injected with UM-SCC-38 overexpressing FL or FLm (Fig. 3F). Similar to the CAM model, tumors overexpressing E6*I or control (PCD) exhibited invasive islands further from the main tumor than tumors overexpressing FLm, but not FL (expresses low amount of E6*I) (Fig. 3G–3H; S3C). These data show that E6*I promotes an aggressive pattern of invasion *in vivo*, which corroborates *in vitro* findings.

### 5. E6 splicing to E6*I in HPV+ OPSCC promotes aggressive invasion and p-EMT.

In the above approach, UM-SCC-38 stably overexpressed E6 but does not have E7 because it is an HPV(−) OPSCC cell line. This approach allowed investigation of the impact of solely E6 and its isoforms. However, in HPV+ OPSCC, E6 co-operates with E7 (65). Moreover, UM-SCC-38 contains a homozygous missense mutation G396T in p53 (66), whereas HPV+ OPSCC typically has wild type p53 (67). Therefore, we also verified the role of HPV E6 isoforms in HPV+ OPSCC cell lines with E6, E7, and wild-type p53 (65).

**Fig. 4:**
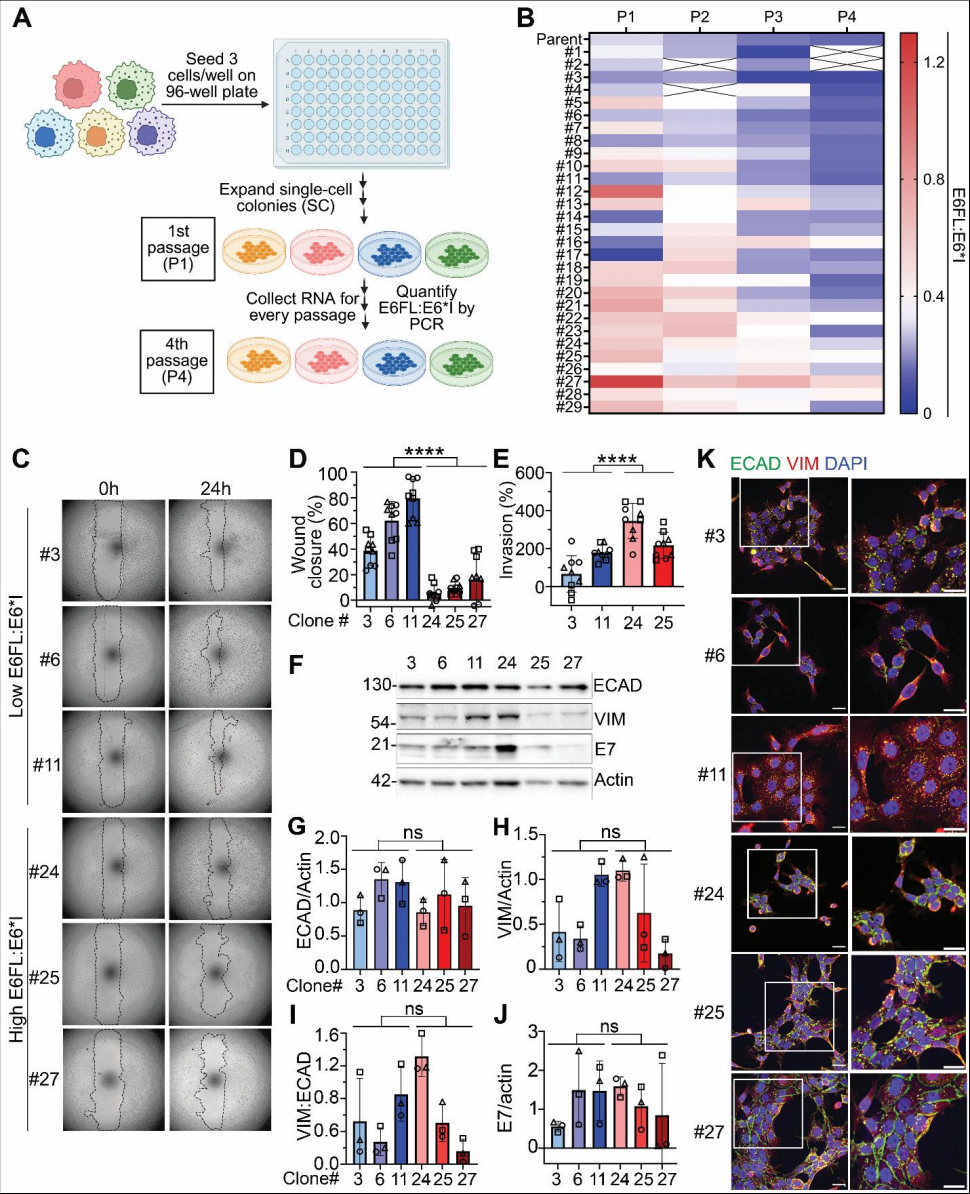
Low E6FL: E6*I is associated with increased migration in UPCI:SCC154 single-cell clones. (A) Schematic illustrating single-cell (SC) clone isolation and expansion. Created with Biorender.com. (B) Heat map showing the values of E6FL:E6*I for each clone. X indicates that expression of E6FL or E6*I was undetectable. (C) Representative images for wound healing assays from three independent experiments. (D) Migration is quantified from (C) and represents percent (%) wound closure after 24 h. (E) Fluoroblok transwell invasion assay was performed. Invasion was calculated as a percent of migrated cells (see Fig,S4B). Each shape is an independent experiment (n=3), with three replicates per experiment. (F) Representative immunoblot of ECAD and VIM in UPCI:SCC154SC clones. Actin was used as a loading control. (G-J) Densitometry of ECAD (G) andvimentin (H),were normalized to actin. VIM:ECAD ratio (I) was quantified to assess mesenchymal phenotype. Densitometry of E7 (J) was normalized to actin. Each shape represents an independent experiment (n=3); ns= non-significant (unpaired t test). (K) Immunofluorescence staining of DAPI (blue), ECAD (green) and VIM (red) in SC clones. Individual channels are shown in Fig. S4F. Right panel shows enlarged images from boxed area in left panel Scale bar = 100um (both left and right). Data in bar graphs are represented as mean ± SD. (*p<0.05, ** p<0.01, ****p<0.0001 unpaired t test).

Since cell lines exhibit intrinsic heterogeneity (44), we hypothesized that parent UPCI:SCC154 can yield single cell-derived (SC) clones with varying E6FL:E6*I. We isolated 29 SC clones from UPCI:SCC154 and investigated expression of E6 isoforms, presented as E6FL:E6*I ratio (Fig. 4A,4B). Low E6FL:E6*I indicates increased splicing of E6FL to E6*I, and vice versa. As expected, the 29 clones had a range of E6FL:E6*I across 4 consecutive passages; in later passages, E6FL:E6*I was progressively more homogeneous between some clones (Fig. 4B). Further studies focused on SC clones with stable low (#3, 6, 11) or higher (#24, 25, 27) E6FL:E6*I over 4 consecutive passages (Fig. 4B). Functional assays were performed only within the first four passages. UPCI:SCC154SC clones with low E6FL:E6*I had significantly increased growth, migration and invasion than clones with high E6FL:E6*I (Fig. 4C–4E; S4A, S4B). At 14-15 days, colonies from #3 and #11 (low E6FL:E6*I) had a more diffuse distribution (more mesenchymal phenotype) than #24, #25 and #27 (high E6FL:E6*I), which were more cohesive (epithelial phenotype) (Fig. S4C). These phenotypic differences between clones prompted an investigation of EMT markers. ECAD and VIM expression were variable and not significantly different across clones by immunoblot analysis (Fig. 4F–4H). The VIM:ECAD ratio that is commonly used to assess EMT in cancer cells (68), did not differ significantly between clones with low and high E6FL:E6*I (Fig. 4I). Moreover, though E6*I has been shown to increase translation of E7 (69, 70), other studies reported conflicting findings (71, 72). Therefore, we investigated E7 expression in the SC clones by immunoblot (Fig. 4F, 4J). Since E7 degrades Rb and enhances p16 expression, we also investigated Rb and p16 to assess E7 activity (Fig. S4D). Protein expression of E7, Rb and p16 was variable and did not differ significantly between clones with low and high E6FL:E6*I (Fig. 4F, 4J. S4D–S4F). To assess if subcellular localization of ECAD and VIM differed between clones, immunofluorescence was performed (Fig. 4K; Fig. S4G). Clones #3, #6, #11 (low E6FL:E6*I) demonstrated less membrane localization and higher cytoplasmic localization of ECAD than #24, #25, #27 (high E6FL:E6*I) (Fig.4K; S4G), similar to earlier findings with overexpression of E6* or E6FL, respectively (Fig. 2K), which is consistent with p-EMT (36–38). Thus, SC clones isolated from UPCI:SCC154, a HPV+ OPSCC cell line, also support that increased splicing of E6FL to E6*I is associated with enhanced migration and p-EMT.

**Fig. 5:**
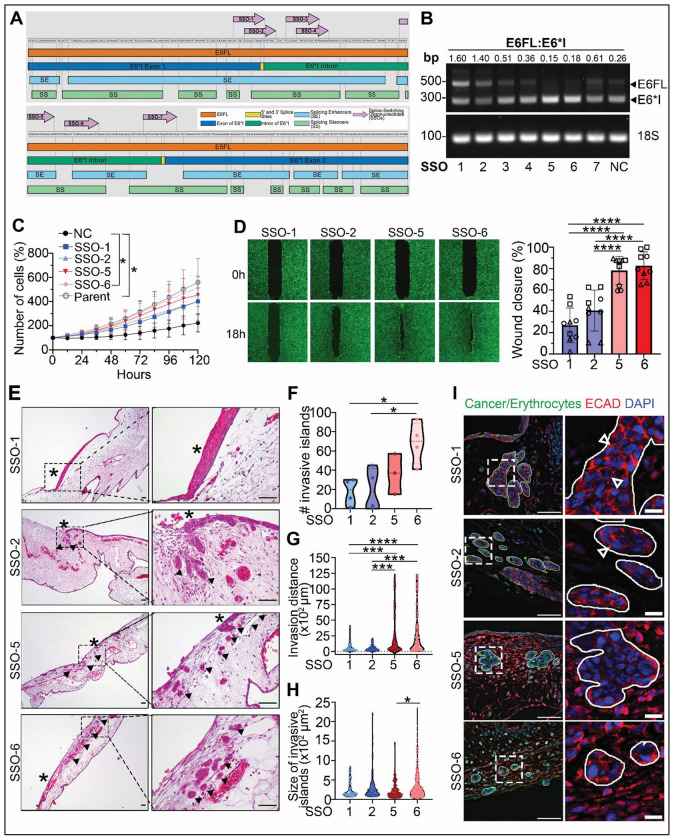
Inhibition of E6FL splicing suppresses invasion of HPV16+ OPSCC. (A) Schematic illustrating position of SSOs (purple) relative to splicing enhancer and splicing silencer sequences in HPV16 E6 pre-mRNA. (B) Representative agarose gel from 3 independent experiments validating expression of E6 isoforms in cDNA of UPCI:SCC154 transiently transfected with SSOs. E6FL:E6*I ratios were measured by densitometry. (C) Proliferation assays. Viable cells expressed as percent (%) of 0h. (D) Representative images for wound healing assays. Cells were stained with CellTracker^™^ Green CMFDA Dye. Bar graph shows migration, represented as percent (%) wound closure after 18h. Each shape represents an independent experiment (three); each point represents one replicate. At least three replicates were quantified per experiment. (E) Representative H&E images from CAM assay. UPCI:SCC154 transfected with SSOs were grafted on the upper CAM, which was harvested 3 days later, and stained. Arrowheads indicate some of the invasive tumor islands. Asterisk indicates main tumor bulk. Scale bar = 100μm. 3-6 eggs/group. (F-H) Quantification of invasive islands from CAM assay in (E). Number of invasive islands (F), nearest distance from invasive islands to the tumor bulk (G), and size of invasive islands (H) were quantified from the entire CAM section. Each point in (F) represents a different egg. Each point in (G) and (H) represents an invasive tumor island. Data are represented as mean ± SD. *p<0.05, **p<0.01, ***p<0.001, ****p<0.0001 (One-way ANOVA post-hoc Tukey test). (I) Immunofluorescence staining of ECAD (red) in tumor islands (outlined in white) of upper CAM; cancer in green; DAPI in blue. Right panel is enlarged for boxed area in left panel. White arrowheads point to examples of membrane ECAD localization. Scale= 50μm (left); 10μm right).

The above studies in HPV16+ OPSCC support an association between low E6FL:E6*I ratio and spread. To directly investigate if inhibition of E6 splicing in the presence of E7 prevents progression of HPV16+ OPSCC, we used antisense splice-switching oligonucleotides (SSOs). SSOs are short, synthetic nucleic acids that base pair with a pre-mRNA to disrupt splicing by blocking RNA-RNA or protein-RNA interactions with splicing machinery; they have been used to treat multiple disorders such as spinal muscular atrophy (73). We designed seven SSOs to target splice sites or splice enhancer/silencer regions of E6 pre-mRNA to inhibit splicing of E6FL into E6*I (Fig. 5A). The impact of these SSOs on HPV16 E6 splicing was screened in UPCI:SCC154 (Fig. 5B), which has high intrinsic E6 splicing (Fig. 1B). SSO-1 and SSO-2 increased E6FL:E6*I more than five-fold compared to control non-targeting SSO (NC), supporting that these two SSOs block splicing (Fig. 5B). In contrast, SSO-5 and SSO-6 slightly reduced E6FL:E6*I relative to NC (Fig. 5B). In functional studies, cells transfected with SSO-1 and SSO-2 (high E6FL:E6*I) had no significant difference from SSO-5 and SSO-6 (low E6FL:E6*I) (Fig. 5C). The growth rate of cells transfected with NC SSO was minimal compared to untransfected parent cells suggesting inherent toxicity of NC SSO (Fig. 5C).Therefore, we compared SSO-1 and SSO-2 (high E6FL:E6*I) to SSO-5 and SSO-6 (low E6FL:E6*I) for functional assays. SSO-1 and SSO-2 significantly decreased wound closure compared to SSO-5 and SSO-6 (Fig. 5D).

To investigate if blocking E6FL splicing abrogated aggressive invasion *in vivo*, we grafted UPCI:SCC154 transfected with SSOs on the CAM (Fig. 5E). After 3 days, tumors with low E6FL:E6*I (SSO-5, SSO-6) showed more invasive islands than tumors with high E6FL:E6*I (SSO-1, SSO-2); SSO-6 was significantly different compared to SSO-1 and SSO-2 (Fig. 5F). Invasive islands in tumors with SSO-5 and SSO-6 were significantly further from the main tumor than SSO-1 and SSO-2 (Fig. 5G). There was no significant difference in size of islands between tumors with high versus low E6FL:E6*I (Fig. 5H).

Since increased splicing of E6FL to E6*I promoted cytoplasmic localization of ECAD (Fig. 2K; 4K), we validated expression of ECAD in tumor cells from the CAM assay (Fig. 5I). Invasive islands from tumors with SSO-1 and SSO-2 (high E6FL:E6*I) showed higher membrane ECAD expression than SSO-5 and SSO-6 (low E6FL:E6*I) (Fig. 5I). Findings from this orthogonal approach in HPV+ OPSCC cells (UPCI:SCC154) corroborate *in vitro* and *in vivo* results with overexpression of solely E6 and its isoforms in HPV(−) OPSCC cells (UM-SCC-38), supporting the important role of E6*I in driving aggressive invasion in HPV16+ OPSCC, regardless of the presence of E7.

### 6. E6*I induces endocytosis of E-cadherin.

**Fig. 6:**
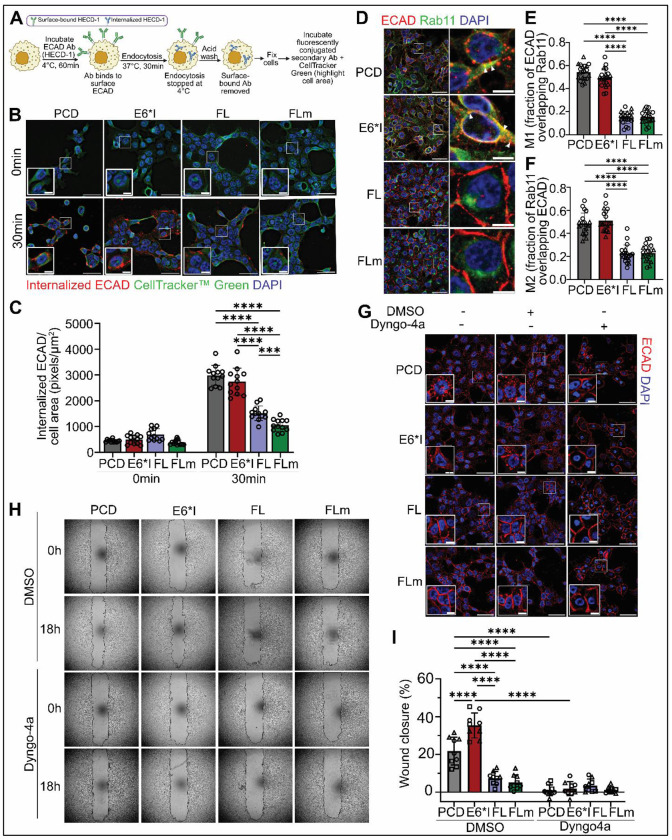
E6*I promotes endocytosis of ECAD to induce migration in OPSCC (A) Schematic diagram of antibody internalization assay. (B) Representative immunofluorescence images of antibody internalization assay performed on UM-SCC-38 overexpressing PCD or E6 isoforms. Red = internalized ECAD, green = CellTracker^™^ Green (whole cells). Blue = DAPI (nucleus). Enlarged images of boxed areas are showed in lower left. Scale Bars: Outside=50μm, inside=10μm (C) Quantification for (B). Fluorescence intensity of internalized ECAD (red) are quantified within cell areas marked by CellTracker^™^ Green. *** p<0.001; **** p<0.0001 (two-way ANOVA) (D) Maximum projection of images showing co-localization of ECAD (red) and Rab11 (green) in UM-SCC-38 expressing PCD control or E6 isoforms. DAPI (nucleus) is in blue. Right panel shows enlarged image of boxed area in left panel. Scale bar= 50μm (left) and 10μm (right). (E&F) Co-localization of ECAD and Rab11 was quantified from 12 z slices, 5 fields/duplicate. Manders’ coefficients, i.e., M1, fraction of ECAD overlapping Rab11 (E) and M2, fraction of Rab11 overlapping ECAD (F), were quantified using ImageJ JaCoP plugin. **** p<0.0001 (one-way ANOVA post-hoc Tukey). (G) Immunofluorescence on UM-SCC-38 overexpressing PCD or E6 isoforms treated with either Dyngo-4a or DMSO vehicle. Cells were fixed 24h after treatment and stained with ECAD (red) and DAPI (blue). Enlarged images of boxed areas are showed in lower left. Scale Bars: Outside=50μm, inside=10μm. (H) Wound healing assay for UM-SCC-38 cells overexpressing PCD control or E6 isoforms treated with either Dyngo-4a or DMSO vehicle. (I) Migration is quantified from (H) and represents percent (%) wound closure after 18h.

Turnover and endocytosis of epithelial proteins are key mechanisms that control junctional stability and induce epithelial-mesenchymal plasticity, which are essential for cancer spread (74). Since OPSCC cells overexpressing E6*I have higher cytoplasmic and lower membrane localization of ECAD compared to FL and FLm (Fig. 2K, 4K, 5I), we hypothesized that E6*I induces endocytosis of ECAD to enhance spread. To test this hypothesis, we first visualized ECAD internalization with an antibody against its extracellular region (HECD-1) in UM-SCC-38 overexpressing E6 isoforms or control cells based on an established protocol (75) (Fig. 6A). We observed that cells overexpressing E6*I had more internalized ECAD after 30 min of endocytosis compared to E6FL and E6FLm; while control (PCD) cells had similar internalized ECAD as E6*I (Fig. 6B, 6C). Since sequestration of proteins into vesicles is an important step for endocytosis (74), we investigated co-localization of ECAD with EEA1 or Rab11, markers for early and recycling vesicles, respectively. Co-localization of ECAD and EEA1 did not change significantly between E6 isoforms (Fig. S5A–S5C). In contrast, ECAD was co-localized with Rab11 more prominently in cells with E6*I or PCD compared to FL and FLm (Fig. 6D–6F; S5D). This shows that turnover of surface ECAD in E6*I-overexpressing UM-SCC-38 during p-EMT is associated with storage in Rab11-positive recycling endocytic vesicles, consistent with previous studies in pancreatic cancer and non-cancer models (36, 58, 76).

To investigate if endocytosis of ECAD is critical for E6*I-induced migration, we inhibited endocytosis with Dyngo-4a, a potent inhibitor for dynamin-mediated endocytosis (77, 78). Dyngo-4a enhanced membrane ECAD expression in cells overexpressing E6*I, consistent with blocking endocytosis (Fig. 6G). In cells overexpressing FL or FLm, ECAD was mostly localized at the membrane regardless of Dyngo-4a treatment (Fig. 6G), suggesting that less endocytosis of ECAD occurred in these cells (Fig. 6A–6C). Dyngo-4a significantly reduced wound closure in cells overexpressing E6*I, but not in cells overexpressing FL and FLm (Fig. 6H, 6I), consistent with their membrane ECAD expression (Fig. 6G). These results support that endocytosis of ECAD is a key mechanism regulating E6*I-driven invasion in OPSCC.

### 7. Patients with lower E6FL:E6_ALL_ have lower membrane:cell ECAD, which correlates with poor outcomes

**Fig. 7:**
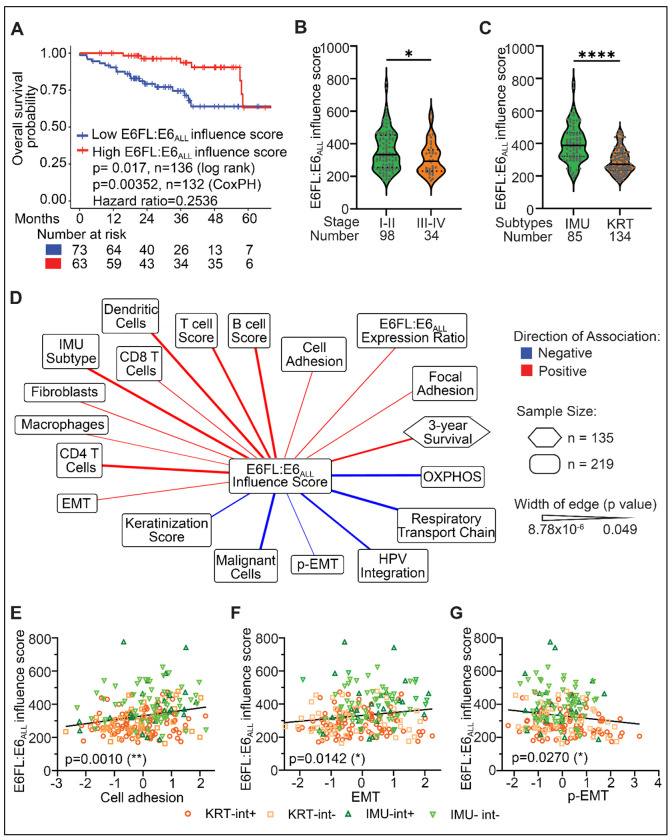
Low E6FL:E6_ALL_ influence scores correlate with worse disease outcomes and p-EMT in human HPV+ OPSCC. (A) Kaplan-Meier curve showing significant segregation of overall survival between high and low E6FL:E6_ALL_ Influence score. High vs. low E6FL:E6_ALL_ Influence score was determined by patients above or below the mean of E6FL:E6_ALL_ Influence score (331.24 logCPM). Log rank and Coxph model p values (account for cohort, stage and age) are provided. (B&C) Violin plots showing E6FL:E6_ALL_ influence scores in patients segregated by overall stage (B) and molecular subtype (C). *p<0.05; **** p<0.0001 (unpaired t test). (D) Network graph showing only statistically significant associations (p<0.05) with E6FL:E6_ALL_ influence score. Associations were determined with linear regression models (correcting for cohort) for continuous variables, and with logistic regression models (correcting for cohort) for categorical variables. (E-G) Scatter plots of cell adhesion (E), EMT (F) and p-EMT (G) gene scores by E6FL:E6_ALL_ Influence score. Each point represents one patient and is classified according to molecular subtype (KRT or IMU) and HPV integration status (integration-positive (int+) or integration-negative (int−)).

Having established that E6FL and E6*I have differential roles in invasion in multiple *in vitro* and *in vivo* models, we investigated their impact in patients. In our previous study, we generated an E6FL:E6_ALL_ influence score, in 68 HPV+ HNSCC (>75% OPSCC), where E6_ALL_ represents all variants of E6 including E6* and E6FL (32). The influence score was derived from 168 genes that significantly correlated with E6FL:E6_ALL_ transcript expression (32). High E6FL:E6_ALL_ influence score suggests higher impact of E6FL, while low E6FL:E6_ALL_ influence score suggests higher impact of E6* (32). We validated the clinical relevance of E6FL:E6_ALL_ influence score by expanding our analysis to 219 HPV+ HNSCC (199 OPSCC and 20 non-OPSCC) from four patient cohorts (details of anatomic sites and HPV genotypes in Tables S1 and S2, respectively). This is the largest cohort to date used to validate the impact of E6 splicing in patients. Using the 136 of 219 patients with survival data, lower E6FL:E6_ALL_ influence score, segregated by the mean, correlated with worse overall survival (Fig. 7A) and more advanced overall stage (Fig. 7B), but not with T or N stage when thesewere considered separately (Fig. S6A, S6B). Previously, we identified two subtypes for HPV+ OPSCC, namely KRT (highly keratinized) and IMU (immune strong); KRT has worse prognosis than IMU (28). Analysis in 219 HPV+ HNSCC demonstrated that E6FL:E6_ALL_ influence score was significantly reduced in patients classified as KRT (more aggressive tumors) (Fig. 7C). Lower E6FL:E6_ALL_ influence score was observed in OPSCC with HPV integration (Fig. S6C), a feature commonly associated with worse clinical outcomes (7, 79). These findings in patients support pre-clinical data where increased splicing to E6* promotes OPSCC progression.

To understand the impact of E6 splicing, we independently investigated the correlation of E6FL:E6_ALL_ influence score with clinical variables and gene signatures related to cancer progression. For categorical associations, including associations with HPV integration status, and molecular subtype, logistic regression was employed with cohort as a covariate. For all continuous associations (such as immune cell score), a linear model was used with cohort as a covariate. We observed that E6FL:E6_ALL_ influence score positively correlated with 3-year overall survival and the IMU subtype (Fig. 7D), that is associated with less aggressive tumors (28). E6FL:E6_ALL_ influence score negatively associated with HPV integration status, a prognostic factor that may predict poorer survival (7, 79–81). Together, these findings support the prognostic significance of the E6FL:E6_ALL_ influence score.

Since pre-clinical findings suggested that E6*I promotes invasion compared to E6FL (Fig. 2G–2J; 4C–4E), we explored whether the E6FL:E6_ALL_ influence score is associated with gene signatures from related biological processes. Consistent with the epithelial phenotype observed in OPSCC cells with high E6FL (Fig. 2K; 4K), E6FL:E6_ALL_ influence score associated positively with cell adhesion (Fig. 7D, 7E; Table S3). Surprisingly, E6FL:E6_ALL_ influence score also correlated positively with complete EMT (Fig. 7D, 7F), which is conventionally associated with increased invasion and poorer prognosis (82, 83). To explore this further, we investigated p-EMT that has been associated with poor prognosis in HNSCC (56, 84). Using the established p-EMT signature (56, 84), we observed that E6FL:E6_ALL_ influence score negatively correlated with p-EMT (Fig. 7D, 7G). This is consistent with *in vitro* findings where E6*I promotes relocation of membrane ECAD to the cytoplasm (Fig.2K; 4K; 5I), rather than suppressing ECAD expression (Fig. 2L, 2M; 4F, 4G). Previous studies associated reduced oxidative stress and heightened immune response with improved prognosis in OPSCC (7, 28, 34, 51, 85–89). Our analysis demonstrated that E6FL:E6_ALL_ influence score is directly correlated with immune infiltration and negatively with oxidative phosphorylation and respiratory electron transport chain (Fig. 7D; Table S3), which is consistent with a previous study on a smaller cohort (32). This suggests that splicing of E6FL into E6*I may impact immune infiltration and oxidative stress in OPSCC.

**Fig. 8:**
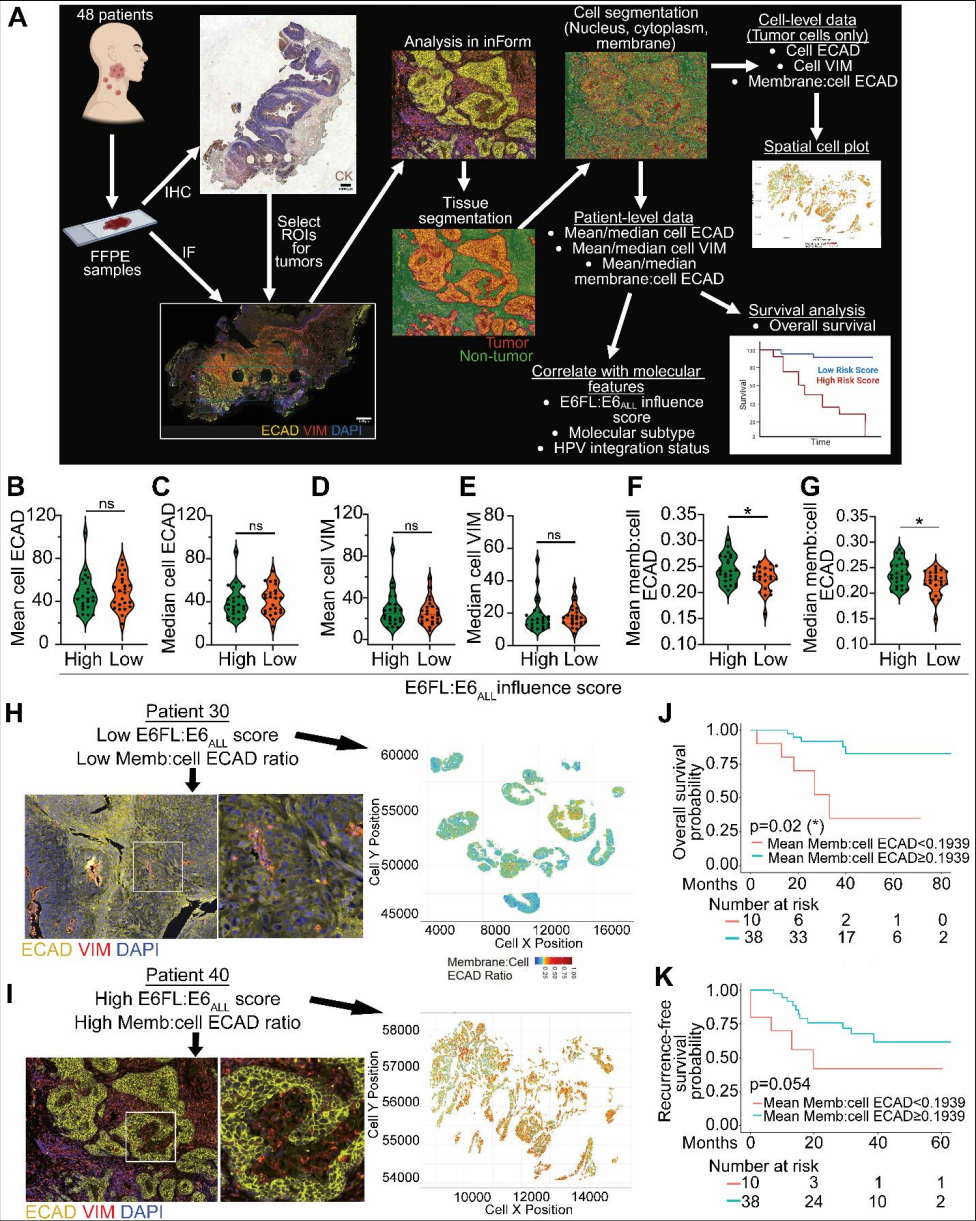
Patients with low E6FL:E6_ALL_ influence score exhibited lower membrane:cell ECAD ratio, which correlates with worse survival. (A) Schematic of multiplexed immunofluorescence and morphometric quantification in 48 human HPV+ OPSCC. (B-G) Mean and median ECAD (B&C), VIM (D&E) and membrane:cell ECAD (F&G) for patients segregated according to high and low E6FL:E6ALL influence score. (H&I) Representative of patient segregated in low (H) and high (I) E6FL:E6ALL influence score. Representative image of region of interest showing ECAD (yellow), VIM (red) and DAPI (blue) is shown (right panel is enlarged boxed area from left panel). The membrane:cell ECAD from segmented tumor cells in each patient was represented in a spatial cell plot according to its X and Y position. (J&K) Overall (J) and recurrence-free (K) survival showing segregation of patients with low and high membrane:cell ECAD (cut-off=0.1939). Log-rank test was performed to determine statistical significance.

We validated the impact of E6FL:E6_ALL_ influence score on p-EMT by independently performing immunofluorescence against ECAD (epithelial) and VIM (mesenchymal) on pre-treatment biopsies from 48 randomly selected patients with HPV+ OPSCC (Fig. 8A). Serial formalin-fixed paraffin-embedded (FFPE) sections were immunohistochemically stained with cytokeratin to highlight epithelial cells (Fig. 8A). Expression of ECAD and VIM were quantified in cells identified as the tumor using Akoya InForm software (Fig. 8A). As expected, mean and median cell ECAD and VIM expression of tumor cells in patients with low E6FL:E6_ALL_ influence score did not differ significantly from those with high E6FL:E6_ALL_ influence score (Fig. 8B–8E). This corroborates our *in vitro* data where expression of ECAD and VIM did not differ significantly in cells with differing E6FL:E6*I ratios (2L, 2M; 4F, 4G).

Since lower E6FL:E6_ALL_ influence score directly correlated with p-EMT signature in our RNA sequencing analysis (Fig. 7D, 7G), we investigated if patients with increased splicing of E6FL into E6*I (i.e., lower E6FL:E6_ALL_ influence score) would exhibit reduced membrane:cell ECAD, which is characteristic of p-EMT and correlates with enhanced ECAD endocytosis. The mean and median membrane:cell ECAD were significantly reduced in tumors with low versus high E6FL:E6_ALL_ influence score (Fig. 8F–8I). This agrees with our in vitro findings (Fig. 2K; 4K; 5I). In contrast, the mean and median ECAD, VIM and membrane:cell ECAD expression did not differ significantly with patients with differing HPV integration status or molecular subtype (Fig. S6D–S6E).

Since E6FL:E6_ALL_ influence score correlates negatively with patient survival and p-EMT, we investigated if membrane:cell ECAD ratio can predict patient prognosis. Patients with lower membrane:cell ECAD was significantly associated with worse overall survival (Fig. 8J) and trended towards worse recurrence-free survival (Fig. 8K). In contrast, mean cell ECAD or VIM expression did not significantly correlate with overall or recurrence-free survival (Fig. S6F–G). This suggests that decreased membrane localization of ECAD, a downstream effect of E6*I, is prognostic in OPSCC, rather than total ECAD or VIM expression.

Overall, our results suggest that high E6FL:E6_ALL_ influence score (i.e., reduced E6 splicing) predicts better prognosis in HPV+ OPSCC and inversely correlates with p-EMT, as observed from RNA sequencing and immunofluorescence analysis. Moreover, membrane:cell ECAD ratio is associated with better prognosis, and may be used as a surrogate marker for E6 splicing in pre-treatment biopsies.

Taken together, these data show that E6FL and E6*I have distinct roles in OPSCC progression. Enhanced splicing of E6FL to E6*I is critical for promoting endocytosis-mediated translocation of ECAD from the cell membrane to the cytoplasm, thereby minimizing cell adhesion and increasing spread of OPSCC cells. These data support that the E6FL:E6*I ratio or membrane:cell ECAD ratio are potential biomarkers to stratify patients for personalized treatment and suggest reversing E6FL splicing as a strategy to treat aggressive HPV+ OPSCC.

## Discussion

One-third of HPV+ OPSCCs behave aggressively but the underlying mechanisms are unclear. Multiple clinical trials to de-escalate treatment for HPV+ OPSCC have been unsuccessful, emphasizing that biomarkers for risk stratification are urgently needed. Here, using orthogonal *in vitro* and pre-clinical models that mimic OPSCC biology, we demonstrate that splicing of full length E6 to shorter isoform E6*I drives aggressive invasion, promotes p-EMT through endocytosis of ECAD, and associates with poorer clinical outcomes. Moreover, targeting E6 splicing by SSOs could mitigate aggressive behavior. This is the first study to establish E6 splicing as a key mechanism driving aggressive behavior in HPV+ OPSCCs and suggest membrane:cell ECAD ratio as a biomarker for risk stratification. Future studies could employ these models to comprehensively investigate other underlying mechanisms in OPSCC.

Increased splicing of E6FL to E6*I induces aggressive invasion *in vivo*, where clusters of tumor cells with higher E6*I spread further from the main tumor than those with reduced splicing. This corresponds to an aggressive clinical presentation of HPV+ OPSCC, such as spread to regional lymph nodes (13, 15, 52, 53). Mechanistic studies in cell lines suggested that E6*I increased cytoplasmic localization of ECAD via endocytosis thereby reducing cell-cell adhesion, which is consistent with p-EMT. Our findings in patients and cell lines suggest that E6*I drives p-EMT; cells with higher splicing of E6FL to E6*I had less membrane ECAD than those with less splicing of E6FL, suggesting that E6*I reduced cell-cell adhesion (57, 58) (36, 37); total ECAD protein expression was unaltered. This was supported by RNA sequencing in multiple patient cohorts correlating E6FL:E6_ALL_ influence score, which assesses the downstream impact of E6 splicing, with cell adhesion, p-EMT, and higher overall stage. Independent validation using immunofluorescence on 48 randomly selected patients supported that tumors from patients with lower E6FL:E6_ALL_ influence score (higher E6* activity) had reduced membrane:cell ECAD ratio .

Importantly, lower membrane:cell ECAD predicted worse overall survival and trended toward poorer recurrence-free survival in patients. Globally, immunohistochemistry (IHC) is widely used for tumor diagnosis. In contrast to more expensive or technically challenging approaches such as in situ hybridization or sequencing, IHC is a cost-effective, technically feasible, easily interpretable diagnostic test with a fast turnaround time (~1 day). These factors are particularly important in settings with limited resources (*90, 91*). Our data support that IHC quantification of membrane:cell ECAD could be used as a surrogate marker for E6FL:E6*I (typically detected through ISH and sequencing) to stratify patients by risk.

In our earlier study of 68 patients with HPV+ HNSCC, lower E6FL:E6_ALL_ influence score (high splicing of E6FL to E6*) predicted more locally advanced tumors (32). However, this was not observed in the larger cohort in this study. In functional studies, wild type E6FL enhanced proliferation of OPSCC cells more than E6*I or unspliceable E6FL (E6FLm). We speculate that an interplay between spliced E6*I and E6FL is necessary to induce growth in HPV+ OPSCC; expression of E6*I or unspliced E6FL alone may be insufficient to promote tumor growth. Future studies could explore how E6FL co-operates with E6*I to regulate proliferation.

In this study, SSOs that block splicing of E6FL to E6*I abrogated aggressive invasion in HPV+ OPSCC, suggesting therapeutic potential. SSOs target splice regulatory sites to prevent or enhance splicing of target genes (92), and have been clinically approved to treat certain genetic diseases, and can be designed to treat cancers (73). We observed that while SSO-1 and SSO-2 that target the 5’ splice site, inhibit splicing, SSO-5 and SSO-6 that target the intronic splice regulatory sequence, increase splicing. It is likely that different regulatory sequences within the E6FL pre-mRNA are differentially modulated by splicing machinery to control splicing. Therefore, targeting spliced variants with appropriately designed SSOs may be a feasible strategy to treat HPV+ OPSCC. Assessing off-target and toxicity effects of these SSOs will be important for developing them into therapeutics.

Previously, we showed that lower E6FL:E6_ALL_ influence score (i.e., gene transcripts impacted by E6FL:E6ALL) correlates with worse overall survival (28, 32), using 68 HPV+ HNSCC (majority OPSCC). This was verified in our expanded cohort of 219 HPV+ HNSCC in this study: E6FL:E6_ALL_ activity is reduced in patients with poorer survival and more advanced overall stages. This meta-analysis represents the largest cohort to date used to study the impact of E6 splicing and revealed potential pathways modulated by E6FL and E6*I. Consistent with our previous study, higher E6FL:E6_ALL_ influence score correlates with decreased immune response (28). Another confirmatory finding was that E6FL:E6_ALL_ influence score negatively associates with oxidative phosphorylation (28, 32). Future studies could focus on the roles of E6FL and E6*I on immune responses and metabolism in HPV+ OPSCC.

While E6 and E7 are established drivers of HPV-mediated carcinogenesis, the functional impact of E6 spliced isoforms in OPSCC has been unclear. Although the mechanisms of HPV-modulated OPSCC progression show some overlap with cervical cancer, OPSCC has a unique anatomic location, prolonged time to development, unique clinical progression (7, 31), and a different viral gene expression profile (93), suggesting that the effects of HPV oncogenes differ between diseases. Prophylactic vaccines against high-risk HPVs have been used as an effective strategy to prevent cervical cancer (7). However, the rising incidence of HPV+ OPSCC suggest that the benefit of the HPV vaccine is unlikely to be seen for the next 20-30 years, likely due to the long delay between oral HPV infection and cancer development (~25-30 years) (7, 94). This emphasizes the need to develop effective treatment approaches for HPV+ OPSCC. Our study provides a logical strategy for future therapeutic intervention in HPV+ OPSCC by minimizing splicing of HPV E6.

In conclusion, our comprehensive investigation utilizing multiple approaches to modulate expression of E6*I and E6FL, revealed that splicing of E6FL to E6*I is critical for aggressive invasion in HPV+ OPSCC. Introduction of SSOs to inhibit E6FL splicing, effectively mitigated aggressive invasion. Targeting E6FL splicing with SSOs could be a logical strategy for therapeutic intervention to reduce E6*I in patients with HPV+ OPSCC. Importantly, we elucidated the mechanism by which E6*I promotes aggressive invasion, which lead to identification of a potential biomarker, membrane:cell ECAD ratio that warrants validation in independent cohorts. Together, our findings show that low membrane:cell ECAD ratio in pre-treatment biopsies of HPV+ OPSCC, could stratify patients according to risk; patients with a low ratio may not be candidates for treatment de-intensification trials. Additionally, membrane:cell ECAD ratio could be a clinically scalable biomarker since ECAD is detected by immunohistochemistry, which is widely available in diagnostic pathology laboratories with varying resources.

## Materials and Methods

### Study design

The objective of this study was to identify the mechanistic basis for the disparity between oncogenic effects of HPV16 E6FL and its spliced isoform E6*I in HPV+ OPSCC. Orthogonal approaches to manipulate expression of E6FL and E6*I in vitro were: overexpression, single-cell clone isolation, and SSOs. RT-PCR and RT-qPCR were performed to confirm expression of E6 isoforms. Immunoblot revealed effects on targets of HPV E6 and E7, p53, Rb and p16, and on ECAD and VIM for EMT. Oncogenic phenotypes were determined by proliferation, wound healing and transwell assays. Immunofluorescence revealed cellular localization of ECAD and VIM for EMT. For rigor and reproducibility, in vitro studies (except immunofluorescence) included at least three independent experiments. The number of biological replicates and repeats are stated in each figure. Immunofluorescence was performed once or twice; for each experiment, with capture of at least 4-6 images from each duplicate, for a total of 8-12 images per condition in each experiment. Representative images are shown in figures. Both CAM and mouse experiments were performed to study invasion *in vivo*. For CAM assays, each group had 3-6 eggs. Sample size for CAM was selected by prior experience; no power analysis was performed. Eggs were randomized into groups before grafting cancer cells. For mouse experiments, 50 mice were randomized into 4 groups, with 10-14 mice per group. Number of mice were selected based on power analysis. Investigators (YXL, ML) who assessed the results were not blinded to the intervention. Data collection was stopped at experiment endpoint as stated in Materials and Methods.

Animal studies were performed in compliance with the National Institutes of Health guidelines for animal research and approved by the Institutional Animal Care and Use Committee of the University of Michigan. For patient samples, written informed consents were obtained and the study was approved by the UM Institutional Review Board.

### Cell culture

HOK16B (RRID: CVCL_B405) was obtained from Dr. No-Hee Park and cultured as described (48, 95). UPCI:SCC090 (RRID: CVCL_1899) and UPCI:SCC154 (RRID: CVCL_2230) were purchased from American Type Culture Collection (Manassas, VA, USA; ATCC CRL-3239 and CRL-3241 respectively) and cultured according to ATCC instructions. HOK16B, UM-SCC-38 (RRID: CVCL_7749), UM-SCC-47 (RRID: CVCL_7759) and UM-SCC-104 (RRID: CVCL_7712) were obtained from Dr. Thomas Carey and cultured as described (66). All cell lines were genotyped at the University of Michigan Sequencing Core or GeneCopeia (Rockville, MD, USA). The genotypes for UM-SCC cell lines were validated against published sequences (96, 97). The genotypes for ATCC cell lines were validated with provided sequences.

### Patient cohorts

Four patient cohorts were used in this study, including (1) our previously well-characterized University of Michigan cohort of 18 HPV+ HNSCC (referred to as UM18; GEO #GSE74956) (28), (2) 66 HPV+ HNSCC from The Cancer Genome Atlas (TCGA) (67), (3) 83 HPV+ OPSCC from HPV Virome Consortium (46, 98) (referred to as HVC; European Genome-phenome Archive EGAD00001004366) and (4) our newly introduced UM67 cohort (EGAS50000000893) (99). Details on cohorts, anatomic sites and HPV genotypes are listed in Tables S1 and S2. All other details for UM18, TCGA and HVC have been published (28, 46, 67, 98, 99).

For the UM67 cohort, 67 FFPE tissue blocks were collected from the UM Head and Neck Specialized Program of Research Excellence (SPORE) population; 62 were HPV+ and 10 samples were duplicated patients from UM18, yielding a total of 52 patients for this study (Table S1–S2). Written informed consents were obtained and the study was approved by the UM Institutional Review Board. Patients were enrolled between 2008 and 2014. Epidemiologic, demographic and follow-up information for survival were collected.

### Plasmid construction

E6 plasmids were designed and constructed by the University of Michigan Vector Core. Primers that were used to amplify DNA for subcloning are listed in Table S4. Briefly, polymerase chain reaction (PCR) was performed on pLentiLox HPV16 E6-E7 IRES-PURO to amplify a fragment encoding E6FL using E6-BamHI-F as the forward primer and E6FL-EcoRI-R as the reverse primer. To amplify E6*I, PCR was performed on the same plasmid using E6-BamHI-F as the forward primer and E6*I-EcoRI-R as the reverse primer. PCR fragments were subcloned into the BamHI/EcoRI sites of pcDNA3 vector to generate pcDNA3-E6FL and pcDNA3-E6*I.

To generate pcDNA3-E6FLm, PCR was performed on p1321-HPV16 E6/E7 (Addgene #8641) using E6FLm-BamHI-F and E6-splice-mutant-mega-R as the forward and reverse primers, respectively to generate a megaprimer (100). This PCR product was gel purified and used as a forward primer for megaprimer mutagenesis (100), where E6FLm-EcoRI-R primer was used as the reverse primer. The resulting PCR product containing the splice mutation that changed Val42 to Leucine was cloned into BamHI/EcoRI digested pcDNA3. A silent mutation to Arg40 codon was also observed in the final pcDNA3-E6FLm construct. To generate pcDNA3-E6*I 3’UTR, PCR was performed on p1321-HPV16 E6/E7 using E6*I-3’UTR-BsmGI-F and E6*I-3’UTR-EcoRI-R as forward and reverse primers, respectively. Amplified product was cloned into the BsmGI/EcoRI site of pcDNA3-E6*I to generate pcDNA3-E6*I 3’UTR.

### Cell transfection

For UM-SCC-38, empty vector (pcDNA3), E6*I, E6*I 3’UTR, E6FL and E6FL mutant plasmids were transfected using Amaxa Cell Line Nucleofector kit V (Lonza, Basel, Switzerland, # VCA-1003) with the U20 program according to the manufacturer’s instructions. Geneticin (G418; Gibco) was added to cells for selection of stably transfected cells. UM-SCC-38-pcDNA3, UM-SCC-38-E6*I, UM-SCC-38-E6*I 3’UTR were maintained in 250 μg/ml G418, while UM-SCC-38-E6FL and UM-SCC-38-E6FLm were maintained in 150 μg/ml G418. G418 was removed from culture media for all experiments.

SSOs used in this study were designed by Integrated DNA Technologies (Coralville, IA, USA) and synthesized as 2’-O-methoxyethyl modified oligonucleotides by Genscript (Piscataway NJ, USA). Sequences of SSOs are listed in Table S5. Briefly, cells at 40% confluence were transfected with SSOs using Lipofectamine RNAimax (Invitrogen, Waltham, MA, USA, # 13778075). 24 h after transfection with SSOs, cells were trypsinized and plated for subsequent functional assays.

### Single-cell clone isolation and expansion

UPCI:SCC154 cells were passed through a round bottom polystyrene test tube, with cell strainer snap cap (Corning, Corning, NY, USA) to separate the cell suspension into single cells. Cells were diluted to 30 cells/ml; 100 μl of the cell suspension (3 cells/well) were added to each well in a 96-well plate. 24 hours after attachment, the plate was scanned under a bright-field microscope; wells with one attached cell were recorded. Single cells were maintained in EMEM media with 10% FBS and 100 μg/ml penicillin and streptomycin until growth into a colony (>50 cells), which was expanded from 96- to 24- to 6-well to 100mm plates. Passage 0 was designated when cells reached 50-70% confluence in a 100 mm dish, then split into three; one fraction was seeded in a 100 mm plate (passage 1), the second fraction was frozen, and the third fraction was harvested for subsequent RT-PCR. This process was continued till passages 4 or 5.

### Treatment with Dyngo-4a

For endocytosis inhibition, cells were serum-starved for 2h before they were incubated with 50μm of Dyngo-4a (Abcam ab120689). DMSO was used as a vehicle control. Cells were either subjected to immunofluorescence at 24h after treatment, or wound healing assay after Dyngo-4a was added.

### Reverse transcription-PCR (RT-PCR)

RNA was extracted with the miRNA isolation kit (QIAGEN, Hilden, Germany, # 217084). cDNA was synthesized using SuperScript II reverse transcriptase (Invitrogen #18064014). PCR was performed in the Proflex base PCR System (Applied Biosystems, Waltham, MA, USA) using Taq DNA polymerase (New England Biolabs, Ipswich, MA, USA, # M0273S). PCR products were analyzed on an agarose gel (1.6-2.0% in Tris-Borate-EDTA buffer). Raw images of agarose gels for this study are in Fig. S7–S9. qPCR was performed with Power SYBR Green Master Mix (Applied Biosystems #43-676-59) on a StepOne Plus Real-Time PCR System (Applied Biosystems # 4376600). Data were analyzed by the relative quantification method with normalization to actin. Primers used for PCR and qPCR are listed in Table S6.

### Western blot (Immunoblot) Analysis

Cell cultures at 60-80% confluence were washed with phosphate-buffered saline and lysed in 1% Nonidet P-40 lysis buffer (50 mmol/L Tris-HCl, pH 7.4, 200 mmol/L NaCl, 2 mmol/L MgCl_2_, with protease inhibitors, and 10% glycerol). Lysates were sonicated, centrifuged, and the supernatant collected. Protein concentration was measured by the Bio-Rad protein assay (Bio-Rad, Richmond, CA). Equal amounts of lysates were electrophoresed on 4-20% Tris-Glycine gels (Invitrogen), transferred to nitrocellulose membranes, and immunoblotted with antibodies against p53 (Cell Signaling, #2524; RRID: B_331743), Rb (Cell Signaling, Danvers, MA, USA; #9309; RRID: AB_823629), p53 (Cell Signaling, #2524; RRID: B_331743), p16 (Santa Cruz, Dallas, TX, USA; sc-1661; RRID: AB_628067), Actin (BD Biosciences, Franklin Lakes, NJ; #612656; RRID: AB_2289199), E7 (Santa Cruz sc-65711;) E-cadherin (BD BioSciences, #610182; RRID: AB_397581) and vimentin (Proteintech, Rosemont, IL, USA; #103661-1-AP; RRID: AB_2273020). After overnight incubation with primary antibodies at 4°C, membranes were incubated with horseradish peroxidase (HRP)-conjugated goat anti-rabbit IgG or goat anti-mouse IgG (Cell Signaling; #7074 and #7076, respectively; RRID: AB_2099233 and AB_330924), and visualized with the SuperSignal West-Pico Chemiluminescent substrate (Pierce, #34577) on a ChemiDoc Imaging System (Bio-Rad; RRID: SCR_019684). Band intensities were quantified by ImageJ software (RRID: SCR_003070; National Institute of Health, Bethesda, MD, USA), expressed as arbitrary densitometric units (DU) and normalized to actin. Raw immunoblot images for this study are in Fig. S10.

### Proliferation assay

Cells were seeded at equal densities (3 × 10^4^ for UM-SCC-38, UM-SCC-47, UM-SCC104, UPCI:SCC090, and UPCI:SCC154) on a 24-well plate (4-6 replicates per condition). After overnight attachment, cell cultures were incubated in a BioTek BioSpa 8 Automated Incubator (Agilent Technologies, Santa Clara, CA, USA; RRID: SCR_019728), at 37°C and 5% CO_2_. Cell cultures were transferred from the BioSpa 8 incubator to a Cytation 5 cell imaging multimode reader (RRID: SCR_019732) every 12h. Four high contrast brightfield images were captured per well and image pre-processing was performed to obtain cell number at each time point according to the manufacturer’s instructions.

### Wound healing assay

Cells were grown to 95-100% confluence in triplicate. After 24-48h, cells were treated with mitomycin C (Sigma #M4287) for 2h in serum-free media (0.5ug/ml for UPCI:SCC154; 1ug/ml for UM-SCC-38 ). 48h after seeding, a scratch wound was created using BioTek Autoscratch tool (Agilent Technologies), and cell cultures were incubated in a BioSpa 8 incubator at 37°C and 5% CO_2_. Images of the wound were taken every 4-6h. The area of the wound at the indicated time point was quantified using ImageJ (RRID:SCR_003070; National Institute of Health).

### Fluoroblok migration and invasion assay

Cells were plated for 24-48h and then stained with CellTracker^™^ Green CMFDA Dye (Invitrogen # C7025) for 1.5h. Fluoroblok migration and invasion assays were performed as described (101). Briefly, labelled cells were trypsinized, resuspended in serum-free media, and added to the top chamber of the Fluoroblok transwell insert (8μm pore size, Corning #351152) coated with 50μg/ml Matrigel^™^ (Growth Factor Reduced, Corning, #356230). Inserts, not coated with matrigel, were used for migration. Media with 10% FBS was added to the lower chamber of the transwell insert as a chemoattractant. Relative fluorescence units (RFU) at 492/517 nm were determined in a SpectraMax i3x Multi-Mode Microplate Reader (Molecular Devices, San Jose, CA, USA) using Softmax Pro (Molecular Devices; RRID: SCR_014240) software. Migration was quantified by normalizing to RFU of an uncoated transwell insert (RFU_m_) with the background RFU at 0h (RFU_b_). Invasion was calculated as a percentage of migration, with the formula: (RFU_i_ – RFU_b_)/(RFU_m_ – RFU_b_) × 100, where RFU_i_ represents the RFU in the coated insert.

### In vivo chick chorioallantoic membrane (CAM) and mouse experiments

For overexpression CAM studies, UM-SCC-38 stably transfected with E6*I, FL, FLm, or vector control were stained with CellTracker^™^ Green CMFDA Dye (Invitrogen # C7025) for 2h then grafted on the CAM (day 10), as described (61). For SSO CAM studies, UPCI:SCC154 transfected with SSO-1, SSO-2, SSO-5, or SSO-6 for 24h, were stained with CellTracker^™^ Green CMFDA Dye (Invitrogen # C7025) and grafted on the CAM (day 10) (61). CAMs were harvested at day 13 and sectioned. Slides were stained with either H&E or immunostained with laminin B1 (Abcam, #ab229025; RRID: AB_3083735) and 4′,6-diamidino-2-phenylindole (DAPI) to highlight basement membrane and nuclei, respectively, as previously described (61).

For the mouse experiment, UM-SCC-38 cells stably transfected with PCD (control) or E6 isoforms were injected at the tongue tip in athymic nude mice (Crl:NU(NCr)-*Foxn1^nu^*; Charles River Laboratories; age 6-7 wk, male, n = 10-14/group) at 1x 10^6^ cells/ mouse. Tumors were monitored for 21 days after which mice were euthanized. Tumors were harvested, processed and sectioned. Tissue sections (5μm, FFPE) were stained with H&E or cytokeratin AE1/AE3 mouse monoclonal antibody (EMD Millipore, IHCR2025–6) to highlight epithelium according to our previously established protocol (102).

Whole slide imaging of H&E (CAM and mice) and CK-stained slides (mice) was performed on a Leica Slide Scanner AT2. Fluorescence images for CAM slides were taken on a THUNDER imager (Leica Microsystems, Wetzlar, Germany; RRID: SCR_023794)]. The number and size of invasive tumor islands, and their distance from the main tumor (tumor bulk), were quantified from H&E images using Halo Image analysis platform v3.1 (Indica labs, Albuquerque, NM, USA) or Qupath v0.5.1(103).

### Immunofluorescence

Cells were plated on poly-L-lysine coated glass coverslips for 24-48h, fixed with 4% paraformaldehyde (PFA) and permeabilized with 1% bovine serum albumin (BSA) and 0.2-0.3% TritonX-100 before incubation with primary antibodies. For co-detection of ECAD and VIM, cells were labelled with antibodies to detect VIM (Proteintech, #103661-1-AP; RRID: AB_2273020) and ECAD (BD Biosciences, #610182; RRID: AB_397581) diluted in 1% BSA and 0.3% TritonX-100 overnight at 4°C. Cells were then incubated for 2h at room temperature with goat anti-mouse Alexa Fluor 488 (Invitrogen #A11029; RRID: AB_2534088) and donkey anti-rabbit Alexa Fluor 568 (Invitrogen #A10042; RRID: AB_2534017) diluted in 1% BSA with 0.3% Triton X-100 to detect primary antibodies ECAD and VIM respectively. For detection of ECAD on Dyngo-4a-treated cells, cells were labelled with antibodies against ECAD (BD Biosciences, #610182; RRID: AB_397581) overnight at 4°C, before being incubated with donkey anti-mouse Alexa Fluor 594 (Invitrogen # A32744; RRID: AB_2762826) for 2h at room temperature. For co-detection of ECAD and Rab11 or EEA1, cells were incubated with antibodies against ECAD (BD Biosciences, #610182; RRID: AB_397581) and either EEA1 (Cell Signaling #3288; RRID: AB_2096811) or Rab11 (Cell Signaling #5589; RRID: AB_10693925) overnight at 4°C. Cells were then incubated with goat anti-rabbit Alexa Fluor 488 (Invitrogen # # A11034; RRID: AB_2576217) and donkey anti-mouse Alexa Fluor 594 (Invitrogen # A32744; RRID: AB_2762826) to detect EEA1 or Rab11, and ECAD respectively for 2h at room temperature.

Coverslips were mounted on slides using ProLong^™^ Gold Antifade mounting medium with DAPI (Thermo Fisher Scientific, Waltham MA, USA; #P36931). 4-6 fields per duplicate in each condition were imaged at 63x [with oil immersion using the THUNDER Imager (Leica). For co-detection of EEA1 or Rab11 and ECAD, 12 z-slices of 0.27um thick (default settings) were taken and degree of co-localization was quantified using Just Another Colocalization Plugin on ImageJ.

### Antibody Internalization

Internalization assay was performed based on an established protocol (75). Briefly UM-SCC-38 cells were seeded on poly-L-lysine-coated glass coverslips for 24h. The cell media was changed to binding buffer (Hanks’ balanced salt solution (HBSS) supplemented with 5mM Ca2+ and 50mg/ml BSA) for 1h at 37°C. Cells were then incubated with an antibody against the extracellular domain of ECAD (HECD-1, Invitrogen #13-1700; RRID: AB_2533003) diluted in binding buffer for 1h at 4°C to allow antibodies to bind to surface ECAD. Then cells were washed twice with PBS and incubated with pre-warmed binding buffer for 0 or 30min at 37°C to allow internalization of surface-bound ECAD antibodies. Endocytosis was stopped by immediately transferring cells to 4°C. Cells were washed twice with ice-cold PBS, followed three times with acid wash buffer (0.5M acetic acid with 0.5M NaCl in PBS; 5min per wash) to remove surface-bound ECAD. To visualize internalized ECAD, cells were fixed with 4% PFA, permeabilized with 0.2% TritonX-100, and incubated with CellTracker^™^ Green CMFDA Dye (Invitrogen # C7025) and secondary antibody donkey anti-mouse Alexa Fluor 594 (Invitrogen # A32744; RRID: AB_2762826) diluted in 1% BSA and 0.2% TritonX-100 for 2h at room temperature. Coverslips were mounted on slides using ProLong^™^ Gold Antifade with DAPI and imaged using Thunder Imager (63x oil immersion, 6 fields per duplicate per condition). Signal intensity of internalized ECAD was quantified using ImageJ. Images were split into channels and the green (CellTracker^™^ Green) and red (internalized ECAD) channels were analyzed. Thresholding was performed to create a binary mask on the green channel. The green channel mask was used to generate the cell area of interest to calculate total internalized ECAD (red) intensity within cells. Results were represented as internalized ECAD intensity over cell area.

### Sample acquisition and pre-processing of patient samples

For UM18, TCGA and HVC, clinical and RNA sequencing data were downloaded from respective sources (28, 46, 67, 98). For UM67 cohort, sections from the FFPE blocks were stained with H&E and assessed by a board-certified, head and neck pathologist (JM) for cellularity and necrosis. Tissue was scraped from areas of >70% cellularity in 5μm sections. The DNAstorm/ RNAstorm 2.0 FFPE Combination Kit (Biotium; Fremont CA, USA; #CD508) was used to isolate RNA according to the manufacturer’s instructions. RNA quality was assessed using the 2100 Bioanalyzer System (Agilent); samples with DV200 >20% were selected for sequencing. The QIAseq FastSelect -rRNA HMR+ kit (Qiagen; #334378) was used for library preparation for rRNA-depleted total RNA sequencing. Bulk RNAseq data was generated at the University of Michigan Advanced Genomics Core with NovaSeq S4 (300 cycle and 150bp paired-end). The raw fastq files were aligned to hg38 plus all high-risk HPV genomes with STAR (version 2.7.9a). Quality was checked for all data using fastQC version 0.11.9 (104) (Institut Pasteur, Paris, France). Adapter sequences were trimmed using Cutadapt version 3.4 (105) (National Bioinformatics. Infrastructure Sweden; RRID:SCR_011841). Tumors with more than 200 aligned HPV reads (62 of the 67 samples) were recognized as HPV+. Batch corrections were performed using ComBat-Seq (106). Raw gene counts were converted to log_2_ (cpm) using the edgeR version 3.34.1 cpm function. (107–109), andTPM and logTPM were calculated.

BAM files from the HVC cohort were converted to fastq with samtools version 1.12 (110, 111) and pre-processed in the same way as the UM67 cohort, resulting in 83 HPV+ OPC HVC samples. Ten HPV+ samples from nine unique patients in the UM67 cohort (FFPE) were from patients in UM18 (fresh frozen samples). Duplicates were removed for all analysis.

### E6FL:E6_ALL_ influence scores, E6FL:E6_ALL_ expression ratio and HPV integration status

E6FL:E6_ALL_ expression ratio and E6FL:E6_ALL_ influence scores were computed for the four cohorts as described (32). Integration status was defined as positive for patients having one or more expressed HPV insertion events, detected using SurVirus with default settings (112).

### Survival and staging

To plot Kaplan-Meier survival curves, patients were classified as having either high or low E6FL:E6_ALL_ influence scores, based on the mean. Overall survival was analyzed for the 136 patients for which outcome data is available (UM18, UM67, and TCGA; Table S1–S2). Using the R packages survival and survminer, Kaplan-Meier curves were created, and a p value was calculated using a univariate log-rank test for all 136 patients. A Cox proportional hazards regression model was used in 132 patients (4 removed due to missing data) after adjusting for age, clinical stage, and cohort. TNM staging for all samples was ascertained from medical chart data and reviewed by attending clinicians and converted to overall AJCC tumor stage 8^th^ edition. To generate the violin plots in Fig. 7B, 7C, and S6A–C, E6FL:E6_ALL_ influence scores and E6FL activation scores were plotted against overall stage, T stage, N stage, HPV integration or molecular subtype on Prism Graphpad 10.2.3. Unpaired t test was performed to determine statistical significance between two groups.

### Association tests between E6FL:E6_ALL_ influence scores and molecular pathways

Pathway scores characterizing the tumor immune microenvironment, differentiation state and oxidative phosphorylation were computed across the four cohorts. These scores were derived as sample-wise pathway scores aggregated by the rank of gene expression levels, as previously described (28). Samples were ranked based on the expression levels of each gene. The ranks of all genes within the same pathway were summed for each sample, and the resulting values were centered by mean and scaled by standard deviation across samples to determine the final pathway scores. The following signature scores utilized corresponding Gene Ontology (GO) terms: T cell activation (GO: 0042110), B cell activation (GO:0042113), keratinocyte differentiation (GO: 0030216), mesenchymal cell differentiation (GO:0048762), cell matrix adhesion (GO:0007160), epithelial cell migration (GO:0010632), focal adhesion (KEGG:M7253), respiratory electron transport chain (GO:0022904), and oxidative phosphorylation (KEGG:M19540). For EMT (35) and p-EMT (56) scores, negatively regulated genes were ranked in descending order before summation and scaling. Positively regulated genes were ranked in ascending order before summation, scaling, and addition with negatively regulated genes. Cell type deconvolution utilized CIBERSORTx with single-cell RNAseq HNSCC as the reference signature matrix (113). Due to the distinct origins of the signature matrix and the mixture matrix obtained from single-cell RNAseq and bulk RNAseq platforms, respectively, B-mode batch correction was activated to eliminate technical variations. By default, quantile normalization and absolute mode were disabled. The number of permutations was set to 100, meeting the recommended minimum to achieve a robust deconvolution p-value. The resulting cell type deconvolution matrix included proportions of the following cell types for all inputted samples: T cells CD8, T cells CD4, fibroblasts, macrophages, B cells, malignant cells, mast cells, dendritic cells, myocytes, and endothelial cells.

To generate the association network in Fig. 7D, associations between the E6FL:E6_ALL_ influence score and all other clinical and molecular variables were calculated; only statistically significant (p<0.05) associations are included in the network For categorical associations, including associations with T stage, 3 year survival, HPV integration status, and subtype, logistic regression was employed with cohort as a covariate. For all other associations (which are continuous), a linear model was used with cohort as a covariate. Cytoscape version 3.9.1 (114, 115) was utilized to create the network graph. Statistical details are in Table S3.

### Multiplex immunofluorescence staining and morphometric analysis in patients.

5μm FFPE tumor sections on Superfrost Plus charged slides (Fisher Scientific) were stained with cytokeratin AE1/AE3 mouse monoclonal antibody (EMD Millipore, IHCR2025–6) to highlight epithelium according to our previously established protocol (102). Another section was stained with ECAD and VIM for morphometric analysis. Briefly, sections were incubated at 55-60°C overnight and rehydrated in xylene, followed by 100%, 95%, 70% ethanol and milliQ water. Endogenous peroxidase was quenched with 1% hydrogen peroxide diluted in methanol for 30min at room temperature. Antigen retrieval was performed using 10mM sodium citrate dihydrate (pH 6.0) at 92°C for 15min. Then slides were incubated in blocking solution (5% goat and donkey serum in 0.3% TritonX-100) at room temperature for 1h followed by primary antibodies, VIM (Cell Signaling #5741; RRID: AB_10695459) and ECAD (BD Biosciences, #610182; RRID: AB_397581) diluted in 1% BSA and 0.3% Triton X-100 overnight at 4°C. The next day, slides were incubated with goat anti-mouse Alexa Fluor 568 (Invitrogen A11031;RRID: AB_144696) and goat anti-rabbit Alexa Fluor 647 (Abcam ab150079; RRID: AB_2722623) to detect ECAD and VIM, respectively. Slides were coverslipped with ProLong^™^ Gold Antifade mounting medium with DAPI and scanned using Vectra Polaris by the University of Michigan Research Histology and Immunohistochemistry Core. Whole-slide fluorescence images were analyzed using InForm software (Akoya Biosciences). For each slide, 4-8 regions of interest (ROIs) were marked and trained to segment ROIs into tumor, non-tumor and blank (no tissue) areas. Following this, adaptive cell segmentation was trained using the same ROIs to segment cells into cytoplasm (VIM), membrane (ECAD), and nucleus (DAPI). The tissue and cell segmentation algorithm was applied to analyze whole-slide images, and cell-level morphometric features such as expression of ECAD and VIM in the whole cell, nucleus, membrane and cytoplasm were extracted. This expression was analyzed only in tumor cells as identified in the software. Membrane:cell ECAD ratio was calculated for tumor cells.

### Statistical analysis

For all statistical analysis, a p value of less than 0.05 was considered statistically significant. Statistical comparisons between two groups were performed using unpaired t tests, while comparisons between more than two groups were with one-way ANOVA post-hoc Tukey test. Fisher’s Exact Score test was used to statistically compare the degree of laminin-stained basement membrane degradation in the CAM assay. Statistical tests for Kaplan-Meier curve and bioinformatics analysis are described above.

## Supplementary Material

Supplement 1

## Data Availability

All data is present in this paper or Supplementary Information. Information of UM18 and HVC cohorts were provided in GEO #GSE74956 and European Genome-phenome Archive EGAD00001004366 respectively. The UM67 cohort dataset has been submitted to European Genome-phenome Archive EGAD50000001306.

## References

[1] Racovitan V., Elizabeth G., C. W. Y., N. A. C., Lisa C., Wurzba S., Human papillomavirus (HPV) related oropharyngeal cancers in Canada: A multicenter retrospective cohort study. Human Vaccines & Immunotherapeutics 21, 2486768 (2025). 40264440 10.1080/21645515.2025.2486768 PMC12026042

[2] Mahal B. A., Catalano P. J., Haddad R. I., Hanna G. J., Kass J. I., Schoenfeld J. D., Tishler R. B., Margalit D. N., Incidence and Demographic Burden of HPV-Associated Oropharyngeal Head and Neck Cancers in the United States. Cancer Epidemiology Biomarkers & Prevention 28, 1660–1667 (2019). 10.1158/1055-9965.EPI-19-0038 31358520

[3] Chaturvedi A. K., Anderson W. F., Lortet-Tieulent J., Curado M. P., Ferlay J., Franceschi S., Rosenberg P. S., Bray F., Gillison M. L., Worldwide Trends in Incidence Rates for Oral Cavity and Oropharyngeal Cancers. Journal of Clinical Oncology 31, 4550–4559 (2013). 24248688 10.1200/JCO.2013.50.3870 PMC3865341

[4] Machtay M., Moughan J., Trotti A., Garden A. S., Weber R. S., Cooper J. S., Forastiere A., Ang K. K., Factors Associated With Severe Late Toxicity After Concurrent Chemoradiation for Locally Advanced Head and Neck Cancer: An RTOG Analysis. Journal of Clinical Oncology 26, 3582–3589 (2008). 18559875 10.1200/JCO.2007.14.8841 PMC4911537

[5] Price K. A. R., Nichols A. C., Shen C. J., Rammal A., Lang P., Palma D. A., Rosenberg A. J., Chera B. S., Agrawal N., Novel Strategies to Effectively De-escalate Curative-Intent Therapy for Patients With HPV-Associated Oropharyngeal Cancer: Current and Future Directions. American Society of Clinical Oncology Educational Book, 257–269 (2020). 10.1200/EDBK_280687 32213088

[6] Høxbroe Michaelsen S., Grønhøj C., Høxbroe Michaelsen J., Friborg J., von Buchwald C., Quality of life in survivors of oropharyngeal cancer: A systematic review and meta-analysis of 1366 patients. European Journal of Cancer 78, 91–102 (2017). 28431302 10.1016/j.ejca.2017.03.006

[7] Lim Y. X., D’Silva N. J., HPV-associated oropharyngeal cancer: in search of surrogate biomarkers for early lesions. Oncogene, (2024). 10.1038/s41388-023-02927-9 PMC10873204 38191674

[8] Gillison M. L., Trotti A. M., Harris J., Eisbruch A., Harari P. M., Adelstein D. J., Jordan R. C. K., Zhao W., Sturgis E. M., Burtness B., Ridge J. A., Ringash J., Galvin J., Yao M., Koyfman S. A., Blakaj D. M., Razaq M. A., Colevas A. D., Beitler J. J., Jones C. U., Dunlap N. E., Seaward S. A., Spencer S., Galloway T. J., Phan J., Dignam J. J., Le Q. T., Radiotherapy plus cetuximab or cisplatin in human papillomavirus-positive oropharyngeal cancer (NRG Oncology RTOG 1016): a randomised, multicentre, non-inferiority trial. The Lancet 393, 40–50 (2019). 10.1016/S0140-6736(18)32779-X PMC6541928 30449625

[9] Mehanna H., Robinson M., Hartley A., Kong A., Foran B., Fulton-Lieuw T., Dalby M., Mistry P., Sen M., O’Toole L., Al Booz H., Dyker K., Moleron R., Whitaker S., Brennan S., Cook A., Griffin M., Aynsley E., Rolles M., De Winton E., Chan A., Srinivasan D., Nixon I., Grumett J., Leemans C. R., Buter J., Henderson J., Harrington K., McConkey C., Gray A., Dunn J., Moleron R., McArdle O., Dyker K., Al Booz H., O’Toole L., Cook A., Husband D., Loo V., Soe W., Aynsley E., Sridhar T., Jankowska P., Joseph M., Geropantas K., Vaidya D., Griffin M., Hartley A., Vijayan R., Hwang D., Harrington K., Pettit L., Whitaker S., De Winton E., Rolles M., Brennan S., Sen M., Mendes R., Forster M., Chan A., Evans M., Buter J., Srinivasan D., Foran B., Nankivell P., Bryant J., Sharma N., Spruce R., Brooks J., Batis N., Roques T., Bidmead M., Yang H., Nutting C., Tyler J., Henderson J., Baines H., Gasnier A., Miles E., Clark C., Evans M., Radiotherapy plus cisplatin or cetuximab in low-risk human papillomavirus-positive oropharyngeal cancer (De-ESCALaTE HPV): an open-label randomised controlled phase 3 trial. The Lancet 393, 51–60 (2019). 10.1016/S0140-6736(18)32752-1 PMC6319250 30449623

[10] Yom S. S., Torres-Saavedra P., Caudell J. J., Waldron J. N., Gillison M. L., Xia P., Truong M. T., Kong C., Jordan R., Subramaniam R. M., Yao M., Chung C. H., Geiger J. L., Chan J. W., O’Sullivan B., Blakaj D. M., Mell L. K., Thorstad W. L., Jones C. U., Banerjee R. N., Lominska C., Le Q.-T., Reduced-Dose Radiation Therapy for HPV-Associated Oropharyngeal Carcinoma (NRG Oncology HN002). Journal of Clinical Oncology 39, 956–965 (2021). 33507809 10.1200/JCO.20.03128 PMC8078254

[11] Ang K. K., Harris J., Wheeler R., Weber R., Rosenthal D. I., Nguyen-Tân P. F., Westra W. H., Chung C. H., Jordan R. C., Lu C., Kim H., Axelrod R., Silverman C. C., Redmond K. P., Gillison M. L., Human Papillomavirus and Survival of Patients with Oropharyngeal Cancer. New England Journal of Medicine 363, 24–35 (2010). 20530316 10.1056/NEJMoa0912217 PMC2943767

[12] Fakhry C., Zhang Q., Nguyen-Tan P. F., Rosenthal D., El-Naggar A., Garden A. S., Soulieres D., Trotti A., Avizonis V., Ridge J. A., Harris J., Le Q.-T., Gillison M., Human Papillomavirus and Overall Survival After Progression of Oropharyngeal Squamous Cell Carcinoma. Journal of Clinical Oncology 32, 3365–3373 (2014). 24958820 10.1200/JCO.2014.55.1937 PMC4195851

[13] Billfalk-Kelly A., Yu E., Su J., O’Sullivan B., Waldron J., Ringash J., Bartlett E., Perez-Ordonez B., Weinreb I., Bayley A., Bratman S. V., Cho J., Giuliani M., Hope A., Hosni A., Kim J., Hansen A. R., de Almeida J., Tong L., Xu W., Huang S. H., Radiologic Extranodal Extension Portends Worse Outcome in cN+ TNM-8 Stage I Human Papillomavirus–Mediated Oropharyngeal Cancer. International Journal of Radiation Oncology*Biology*Physics 104, 1017–1027 (2019). 10.1016/j.ijrobp.2019.03.047 30953712

[14] Rosen B. S., Wilkie J. R., Sun Y., Ibrahim M., Casper K. A., Miller J. E., Chotchutipan T., Stucken C. L., Bradford C., Prince M. E. P., Rosko A. J., Malloy K. M., McLean S. A., Chinn S. B., Shuman A. G., Spector M. E., Swiecicki P. L., Worden F. P., Shah J. L., Schonewolf C. A., Chapman C. H., Eisbruch A., Mierzwa M. L., CT and FDG-PET radiologic biomarkers in p16+ oropharyngeal squamous cell carcinoma patients treated with definitive chemoradiotherapy. Radiotherapy and Oncology 155, 174–181 (2021). 33069764 10.1016/j.radonc.2020.10.006 PMC10326862

[15] Ferris R. L., Flamand Y., Weinstein G. S., Li S., Quon H., Mehra R., Garcia J. J., Chung C. H., Gillison M. L., Duvvuri U., O’Malley B. W., Ozer E., Thomas G. R., Koch W. M., Gross N. D., Bell R. B., Saba N. F., Lango M., Méndez E., Burtness B., Phase II Randomized Trial of Transoral Surgery and Low-Dose Intensity Modulated Radiation Therapy in Resectable p16+ Locally Advanced Oropharynx Cancer: An ECOG-ACRIN Cancer Research Group Trial (E3311). Journal of Clinical Oncology 40, 138–149 (2021). 34699271 10.1200/JCO.21.01752 PMC8718241

[16] Castellsagué X., Alemany L., Quer M., Halec G., Quirós B., Tous S., Clavero O., Alòs L., Biegner T., Szafarowski T., Alejo M., Holzinger D., Cadena E., Claros E., Hall G., Laco J., Poljak M., Benevolo M., Kasamatsu E., Mehanna H., Ndiaye C., Guimerà N., Lloveras B., León X., Ruiz-Cabezas J. C., Alvarado-Cabrero I., Kang C.-S., Oh J.-K., Garcia-Rojo M., Iljazovic E., Ajayi O. F., Duarte F., Nessa A., Tinoco L., Duran-Padilla M. A., Pirog E. C., Viarheichyk H., Morales H., Costes V., Félix A., Germar M. J. V., Mena M., Ruacan A., Jain A., Mehrotra R., Goodman M. T., Lombardi L. E., Ferrera A., Malami S., Albanesi E. I., Dabed P., Molina C., López-Revilla R., Mandys V., González M. E., Velasco J., Bravo I. G., Quint W., Pawlita M., Muñoz N., Sanjosé S. d., Xavier Bosch F., t. I. I. H. i. Head, N. C. S. Group, HPV Involvement in Head and Neck Cancers: Comprehensive Assessment of Biomarkers in 3680 Patients. JNCI: Journal of the National Cancer Institute 108, (2016). 10.1093/jnci/djv403 26823521

[17] Agalliu I., Gapstur S., Chen Z., Wang T., Anderson R. L., Teras L., Kreimer A. R., Hayes R. B., Freedman N. D., Burk R. D., Associations of Oral α-, β-, and γ-Human Papillomavirus Types With Risk of Incident Head and Neck Cancer. JAMA Oncology 2, 599–606 (2016). 26794505 10.1001/jamaoncol.2015.5504 PMC4956584

[18] Lechner M., Liu J., Masterson L., Fenton T. R., HPV-associated oropharyngeal cancer: epidemiology, molecular biology and clinical management. Nature Reviews Clinical Oncology 19, 306–327 (2022). 10.1038/s41571-022-00603-7 PMC8805140 35105976

[19] Scheffner M., Werness B. A., Huibregtse J. M., Levine A. J., Howley P. M., The E6 oncoprotein encoded by human papillomavirus types 16 and 18 promotes the degradation of p53. Cell 63, 1129–1136 (1990). 2175676 10.1016/0092-8674(90)90409-8

[20] Martinez-Zapien D., Ruiz F. X., Poirson J., Mitschler A., Ramirez J., Forster A., Cousido-Siah A., Masson M., Pol S. V., Podjarny A., Travé G., Zanier K., Structure of the E6/E6AP/p53 complex required for HPV-mediated degradation of p53. Nature 529, 541–545 (2016). 26789255 10.1038/nature16481 PMC4853763

[21] Boyer S. N., Wazer D. E., Band V., E7 protein of human papilloma virus-16 induces degradation of retinoblastoma protein through the ubiquitin-proteasome pathway. Cancer research 56 20, 4620–4624 (1996). 8840974

[22] Gonzalez S. L., Stremlau M., He X., Basile J. R., Münger K., Degradation of the retinoblastoma tumor suppressor by the human papillomavirus type 16 E7 oncoprotein is important for functional inactivation and is separable from proteasomal degradation of E7. J Virol 75, 7583–7591 (2001). 11462030 10.1128/JVI.75.16.7583-7591.2001 PMC114993

[23] Giarrè M., Caldeira S., Malanchi I., Ciccolini F., Leão M. J., Tommasino M., Induction of pRb Degradation by the Human Papillomavirus Type 16 E7 Protein Is Essential To Efficiently Overcome p16^INK4a^-Imposed G_1_ Cell Cycle Arrest. Journal of Virology 75, 4705–4712 (2001). 11312342 10.1128/JVI.75.10.4705-4712.2001 PMC114225

[24] Arroyo M., Bagchi S., Raychaudhuri P., Association of the Human Papillomavirus Type 16 E7 Protein with the S-Phase-Specific E2F-Cyclin A Complex. Molecular and Cellular Biology 13, 6537–6546 (1993). 8413252 10.1128/mcb.13.10.6537-6546.1993 PMC364713

[25] Roden R. B. S., Stern P. L., Opportunities and challenges for human papillomavirus vaccination in cancer. Nature Reviews Cancer 18, 240–254 (2018). 29497146 10.1038/nrc.2018.13 PMC6454884

[26] Della Fera A. N., Warburton A., Coursey T. L., Khurana S., McBride A. A., Persistent Human Papillomavirus Infection. Viruses 13, (2021). 10.3390/v13020321 PMC7923415 33672465

[27] Olmedo-Nieva L., Muñoz-Bello J. O., Contreras-Paredes A., Lizano M., The Role of E6 Spliced Isoforms (E6*) in Human Papillomavirus-Induced Carcinogenesis. Viruses 10, 45 (2018). 29346309 10.3390/v10010045 PMC5795458

[28] Zhang Y., Koneva L. A., Virani S., Arthur A. E., Virani A., Hall P. B., Warden C. D., Carey T. E., Chepeha D. B., Prince M. E., McHugh J. B., Wolf G. T., Rozek L. S., Sartor M. A., Subtypes of HPV-Positive Head and Neck Cancers Are Associated with HPV Characteristics, Copy Number Alterations, PIK3CA Mutation, and Pathway Signatures. Clinical Cancer Research 22, 4735–4745 (2016). 27091409 10.1158/1078-0432.CCR-16-0323 PMC5026546

[29] Pang J., Nguyen N., Luebeck J., Ball L., Finegersh A., Ren S., Nakagawa T., Flagg M., Sadat S., Mischel P. S., Xu G., Fisch K., Guo T., Cahill G., Panuganti B., Bafna V., Califano J., Extrachromosomal DNA in HPV-Mediated Oropharyngeal Cancer Drives Diverse Oncogene Transcription. Clinical Cancer Research 27, 6772–6786 (2021). 34548317 10.1158/1078-0432.CCR-21-2484 PMC8710294

[30] Walline H. M., Komarck C. M., McHugh J. B., Bellile E. L., Brenner J. C., Prince M. E., McKean E. L., Chepeha D. B., Wolf G. T., Worden F. P., Bradford C. R., Carey T. E., Genomic Integration of High-Risk HPV Alters Gene Expression in Oropharyngeal Squamous Cell Carcinoma. Molecular Cancer Research 14, 941–952 (2016). 27422711 10.1158/1541-7786.MCR-16-0105 PMC5065754

[31] Berman T. A., Schiller J. T., Human papillomavirus in cervical cancer and oropharyngeal cancer: One cause, two diseases. Cancer 123, 2219–2229 (2017). 28346680 10.1002/cncr.30588

[32] Qin T., Koneva L. A., Liu Y., Zhang Y., Arthur A. E., Zarins K. R., Carey T. E., Chepeha D., Wolf G. T., Rozek L. S., Sartor M. A., Significant association between host transcriptome-derived HPV oncogene E6* influence score and carcinogenic pathways, tumor size, and survival in head and neck cancer. Head & Neck 42, 2375–2389 (2020). 32406560 10.1002/hed.26244 PMC8052131

[33] Pang E., Delic N. C., Hong A., Zhang M., Rose B. R., Lyons J. G., Radiosensitization of Oropharyngeal Squamous Cell Carcinoma Cells by Human Papillomavirus 16 Oncoprotein E6∗I. International Journal of Radiation Oncology, Biology, Physics 79, 860–865 (2011). 21106305 10.1016/j.ijrobp.2010.06.028

[34] Sannigrahi M. K., Rajagopalan P., Lai L., Liu X., Sahu V., Nakagawa H., Jalaly J. B., Brody R. M., Morgan I. M., Windle B. E., Wang X., Gimotty P. A., Kelly D. P., White E. A., Basu D., HPV E6 regulates therapy responses in oropharyngeal cancer by repressing the PGC-1α/ERRα axis. JCI Insight 7, (2022). 10.1172/jci.insight.159600 PMC9675449 36134662

[35] Zeisberg M., Neilson E. G., Biomarkers for epithelial-mesenchymal transitions. The Journal of Clinical Investigation 119, 1429–1437 (2009). 19487819 10.1172/JCI36183 PMC2689132

[36] Aiello N. M., Maddipati R., Norgard R. J., Balli D., Li J., Yuan S., Yamazoe T., Black T., Sahmoud A., Furth E. E., Bar-Sagi D., Stanger B. Z., EMT Subtype Influences Epithelial Plasticity and Mode of Cell Migration. Developmental Cell 45, 681–695.e684 (2018). 29920274 10.1016/j.devcel.2018.05.027 PMC6014628

[37] Norgard R. J., Pitarresi J. R., Maddipati R., Aiello-Couzo N. M., Balli D., Li J., Yamazoe T., Wengyn M. D., Millstein I. D., Folkert I. W., Rosario-Berrios D. N., Kim I. K., Bassett J. B., Payne R., Berry C. T., Feng X., Sun K., Cioffi M., Chakraborty P., Jolly M. K., Gutkind J. S., Lyden D., Freedman B. D., Foskett J. K., Rustgi A. K., Stanger B. Z., Calcium signaling induces a partial EMT. EMBO reports 22, e51872 (2021). 34324787 10.15252/embr.202051872 PMC8419705

[38] Pal A., Barrett T. F., Paolini R., Parikh A., Puram S. V., Partial EMT in head and neck cancer biology: a spectrum instead of a switch. Oncogene 40, 5049–5065 (2021). 34239045 10.1038/s41388-021-01868-5 PMC8934590

[39] Parikh A. S., Puram S. V., Faquin W. C., Richmon J. D., Emerick K. S., Deschler D. G., Varvares M. A., Tirosh I., Bernstein B. E., Lin D. T., Immunohistochemical quantification of partial-EMT in oral cavity squamous cell carcinoma primary tumors is associated with nodal metastasis. Oral Oncology 99, 104458 (2019). 31704557 10.1016/j.oraloncology.2019.104458 PMC7382966

[40] Grosse-Wilde A., Fouquier d’Hérouël A., McIntosh E., Ertaylan G., Skupin A., Kuestner R. E., del Sol A., Walters K.-A., Huang S., Stemness of the hybrid Epithelial/Mesenchymal State in Breast Cancer and Its Association with Poor Survival. PLOS ONE 10, e0126522 (2015). 26020648 10.1371/journal.pone.0126522 PMC4447403

[41] Huang R. Y. J., Wong M. K., Tan T. Z., Kuay K. T., Ng A. H. C., Chung V. Y., Chu Y. S., Matsumura N., Lai H. C., Lee Y. F., Sim W. J., Chai C., Pietschmann E., Mori S., Low J. J. H., Choolani M., Thiery J. P., An EMT spectrum defines an anoikis-resistant and spheroidogenic intermediate mesenchymal state that is sensitive to e-cadherin restoration by a srckinase inhibitor, saracatinib (AZD0530). Cell Death & Disease 4, e915–e915 (2013). 24201814 10.1038/cddis.2013.442 PMC3847320

[42] Yu M., Bardia A., Wittner B. S., Stott S. L., Smas M. E., Ting D. T., Isakoff S. J., Ciciliano J. C., Wells M. N., Shah A. M., Concannon K. F., Donaldson M. C., Sequist L. V., Brachtel E., Sgroi D., Baselga J., Ramaswamy S., Toner M., Haber D. A., Maheswaran S., Circulating Breast Tumor Cells Exhibit Dynamic Changes in Epithelial and Mesenchymal Composition. Science 339, 580–584 (2013). 23372014 10.1126/science.1228522 PMC3760262

[43] Lecharpentier A., Vielh P., Perez-Moreno P., Planchard D., Soria J. C., Farace F., Detection of circulating tumour cells with a hybrid (epithelial/mesenchymal) phenotype in patients with metastatic non-small cell lung cancer. British Journal of Cancer 105, 1338–1341 (2011). 21970878 10.1038/bjc.2011.405 PMC3241564

[44] Puram S. V., Mints M., Pal A., Qi Z., Reeb A., Gelev K., Barrett T. F., Gerndt S., Liu P., Parikh A. S., Ramadan S., Law T., Mroz E. A., Rocco J. W., Adkins D., Thorstad W. L., Gay H. A., Ding L., Paniello R. C., Pipkorn P., Jackson R. S., Wang X., Mazul A., Chernock R., Zevallos J. P., Silva-Fisher J., Tirosh I., Cellular states are coupled to genomic and viral heterogeneity in HPV-related oropharyngeal carcinoma. Nature Genetics 55, 640–650 (2023). 37012457 10.1038/s41588-023-01357-3 PMC10191634

[45] Hong A., Zhang X., Jones D., Zhang M., Lee C. S., Lyons J. G., Veillard A.-S., Rose B., E6 viral protein ratio correlates with outcomes in human papillomavirus related oropharyngeal cancer. Cancer Biology & Therapy 17, 181–187 (2016). 26575468 10.1080/15384047.2015.1108489 PMC4847997

[46] Gillison M. L., Akagi K., Xiao W., Jiang B., Pickard R. K. L., Li J., Swanson B. J., Agrawal A. D., Zucker M., Stache-Crain B., Emde A.-K., Geiger H. M., Robine N., Coombes K. R., Symer D. E., Human papillomavirus and the landscape of secondary genetic alterations in oral cancers. Genome Research 29, 1–17 (2019). 30563911 10.1101/gr.241141.118 PMC6314162

[47] Nulton T. J., Olex A. L., Dozmorov M., Morgan I. M., Windle B., Analysis of The Cancer Genome Atlas sequencing data reveals novel properties of the human papillomavirus 16 genome in head and neck squamous cell carcinoma. Oncotarget 8, (2017). 10.18632/oncotarget.15179 PMC5392278 28187443

[48] Park N.-H., Min B.-M., Li S.-l., Huang M. Z., Doniger J. Immortalization of normal human oral keratinocytes with type 16 human papillomavirus. Carcinogenesis 12, 1627–1631 (1991). 1654226 10.1093/carcin/12.9.1627

[49] Pim D., Massimi P., Banks L., Alternatively spliced HPV-18 E6* protein inhibits E6 mediated degradation of p53 and suppresses transformed cell growth. Oncogene 15, 257–264 (1997). 9233760 10.1038/sj.onc.1201202

[50] Antonio-Véjar V., Ortiz-Sánchez E., Rosendo-Chalma P., Patiño-Morales C. C., Guido-Jiménez M. C., Alvarado-Ortiz E., Hernández G., García-Carrancá A., New insights into the interactions of HPV-16 E6*I and E6*II with p53 isoforms and induction of apoptosis in cancer-derived cell lines. Pathology - Research and Practice 234, 153890 (2022). 35487028 10.1016/j.prp.2022.153890

[51] Paget-Bailly P., Meznad K., Bruyère D., Perrard J., Herfs M., Jung A. C., Mougin C., Prétet J.-L., Baguet A., Comparative RNA sequencing reveals that HPV16 E6 abrogates the effect of E6*I on ROS metabolism. Scientific Reports 9, 5938 (2019). 30976051 10.1038/s41598-019-42393-6 PMC6459911

[52] Spector M. E., Chinn S. B., Bellile E., Gallagher K. K., Ibrahim M., Vainshtein J., Chanowski E. J., Walline H. M., Moyer J. S., Prince M. E., Wolf G. T., Bradford C. R., McHugh J. B., Carey T., Worden F. P., Eisbruch A., Chepeha D. B., Matted nodes as a predictor of distant metastasis in advanced-stage III/IV oropharyngeal squamous cell carcinoma. Head Neck 38, 184–190 (2016). 25251643 10.1002/hed.23882 PMC4370799

[53] Ma D. M., Price K., Moore E. J., Patel S. H., Hinni M. L., Fruth B., Foster N. R., Van Abel K., Yin L. X., Neben-Wittich M. A., McGee L. A., Rwigema J. C., Routman D. M., Lester S. C., Price D. L., Janus J. R., Kasperbauer J. L., Nagel T. H., Chintakuntlawar A. V., Savvides P., Garcia J. J., Foote R. L., MC1675, a Phase III Evaluation of De-Escalated Adjuvant Radiation Therapy (DART) vs. Standard Adjuvant Treatment for Human Papillomavirus Associated Oropharyngeal Squamous Cell Carcinoma. International Journal of Radiation Oncology*Biology*Physics 111, 1324 (2021).

[54] Inglehart R. C., Scanlon C. S., D’Silva N. J., Reviewing and reconsidering invasion assays in head and neck cancer. Oral Oncology 50, 1137–1143 (2014). 25448226 10.1016/j.oraloncology.2014.09.010 PMC4254578

[55] Scanlon C. S., Van Tubergen E. A., Inglehart R. C., D’Silva N. J., Biomarkers of Epithelial-Mesenchymal Transition in Squamous Cell Carcinoma. Journal of Dental Research 92, 114–121 (2013). 23128109 10.1177/0022034512467352 PMC3545688

[56] Puram S. V., Tirosh I., Parikh A. S., Patel A. P., Yizhak K., Gillespie S., Rodman C., Luo C. L., Mroz E. A., Emerick K. S., Deschler D. G., Varvares M. A., Mylvaganam R., Rozenblatt-Rosen O., Rocco J. W., Faquin W. C., Lin D. T., Regev A., Bernstein B. E., Single-Cell Transcriptomic Analysis of Primary and Metastatic Tumor Ecosystems in Head and Neck Cancer. Cell 171, 1611–1624.e1624 (2017). 29198524 10.1016/j.cell.2017.10.044 PMC5878932

[57] Le T. L., Yap A. S., Stow J. L., Recycling of E-Cadherin: A Potential Mechanism for Regulating Cadherin Dynamics. Journal of Cell Biology 146, 219–232 (1999). 10402472 PMC2199726

[58] Woichansky I., Beretta C. A., Berns N., Riechmann V., Three mechanisms control E-cadherin localization to the zonula adherens. Nature Communications 7, 10834 (2016). 10.1038/ncomms10834 PMC4792928 26960923

[59] Albergotti W. G., Schwarzbach H. L., Abberbock S., Ferris R. L., Johnson J. T., Duvvuri U., Kim S., Defining the Prevalence and Prognostic Value of Perineural Invasion and Angiolymphatic Invasion in Human Papillomavirus–Positive Oropharyngeal Carcinoma. JAMA Otolaryngology–Head & Neck Surgery 143, 1236–1243 (2017). 29075776 10.1001/jamaoto.2017.2019 PMC5741479

[60] Bauer E., Mazul A., Chernock R., Rich J., Jackson R. S., Paniello R., Pipkorn P., Oppelt P., Gay H., Daly M., El-Mofty S., Thorstad W., Adkins D., Zevallos J., Extranodal extension is a strong prognosticator in HPV-positive oropharyngeal squamous cell carcinoma. The Laryngoscope 130, 939–945 (2020). 31077394 10.1002/lary.28059

[61] Liu M., Scanlon C. S., Banerjee R., Russo N., Inglehart R. C., Willis A. L., Weiss S. J., D’Silva N. J., The Histone Methyltransferase EZH2 Mediates Tumor Progression on the Chick Chorioallantoic Membrane Assay, a Novel Model of Head and Neck Squamous Cell Carcinoma. Translational Oncology 6, 273–281 (2013). 23730406 10.1593/tlo.13175 PMC3660795

[62] Brandwein-Gensler M., Smith R. V., Wang B., Penner C., Theilken A., Broughel D., Schiff B., Owen R. P., Smith J., Sarta C., Hebert T., Nason R., Ramer M., DeLacure M., Hirsch D., Myssiorek D., Heller K., Prystowsky M., Schlecht N. F., Negassa A., Validation of the Histologic Risk Model in a New Cohort of Patients With Head and Neck Squamous Cell Carcinoma. The American Journal of Surgical Pathology 34, 676–688 (2010). 20414102 10.1097/PAS.0b013e3181d95c37

[63] Singh P., Banerjee R., Piao S., Costa de Medeiros M., Bellile E., Liu M., Damodaran Puthiya Veettil D., Schmitd L. B., Russo N., Danella E., Inglehart R. C., Pineault K. M., Wellik D. M., Wolf G., D’Silva N. J., Squamous cell carcinoma subverts adjacent histologically normal epithelium to promote lateral invasion. Journal of Experimental Medicine 218, (2021). 10.1084/jem.20200944 PMC8042603 33835136

[64] Palaniappan T. K., Šlekienė L., Jonasson A.-K., Gilthorpe J., Gunhaga L., CAM-Delam: an in vivo approach to visualize and quantify the delamination and invasion capacity of human cancer cells. Scientific Reports 10, 10472 (2020). 32591581 10.1038/s41598-020-67492-7 PMC7320147

[65] Lim Y. X., Mierzwa M. L., Sartor M. A., D’Silva N. J., Clinical, morphologic and molecular heterogeneity of HPV-associated oropharyngeal cancer. Oncogene, (2023). 10.1038/s41388-023-02819-y PMC10541327 37666939

[66] Nair T. S., Thomas T. B., Yang L., Kakaraparthi B. N., Morris A. C., Clark A. M., Campredon L. P., Brouwer A. F., Eisenberg M. C., Meza R., Carey T. E., Characteristics of head and neck squamous cell carcinoma cell Lines reflect human tumor biology independent of primary etiologies and HPV status. Translational Oncology 13, 100808 (2020). 32574978 10.1016/j.tranon.2020.100808 PMC7317296

[67] Lawrence M. S., Sougnez C., Lichtenstein L., Cibulskis K., Lander E., Gabriel S. B., Getz G., Ally A., Balasundaram M., Birol I., Bowlby R., Brooks D., Butterfield Y. S. N., Carlsen R., Cheng D., Chu A., Dhalla N., Guin R., Holt R. A., Jones S. J. M., Lee D., Li H. I., Marra M. A., Mayo M., Moore R. A., Mungall A. J., Gordon Robertson A., Schein J. E., Sipahimalani P., Tam A., Thiessen N., Wong T., Protopopov A., Santoso N., Lee S., Parfenov M., Zhang J., Mahadeshwar H. S., Tang J., Ren X., Seth S., Haseley P., Zeng D., Yang L., Xu A. W., Song X., Pantazi A., Bristow C. A., Hadjipanayis A., Seidman J., Chin L., Park P. J., Kucherlapati R., Akbani R., Casasent T., Liu W., Lu Y., Mills G., Motter T., Weinstein J., Diao L., Wang J., Hong Fan Y., Liu J., Wang K., Todd Auman J., Balu S., Bodenheimer T., Buda E., Neil Hayes D., Hoadley K. A., Hoyle A. P., Jefferys S. R., Jones C. D., Kimes P. K., Liu Y., Marron J. S., Meng S., Mieczkowski P. A., Mose L. E., Parker J. S., Perou C. M., Prins J. F., Roach J., Shi Y., Simons J. V., Singh D., Soloway M. G., Tan D., Veluvolu U., Walter V., Waring S., Wilkerson M. D., Wu J., Zhao N., Cherniack A. D., Hammerman P. S., Tward A. D., Sekhar Pedamallu C., Saksena G., Jung J., Ojesina A. I., Carter S. L., Zack T. I., Schumacher S. E., Beroukhim R., Freeman S. S., Meyerson M., Cho J., Chin L., Getz G., Noble M. S., DiCara D., Zhang H., Heiman D. I., Gehlenborg N., Voet D., Lin P., Frazer S., Stojanov P., Liu Y., Zou L., Kim J., Sougnez C., Gabriel S. B., Lawrence M. S., Muzny D., Doddapaneni H., Kovar C., Reid J., Morton D., Han Y., Hale W., Chao H., Chang K., Drummond J. A., Gibbs R. A., Kakkar N., Wheeler D., Xi L., Ciriello G., Ladanyi M., Lee W., Ramirez R., Sander C., Shen R., Sinha R., Weinhold N., Taylor B. S., Arman Aksoy B., Dresdner G., Gao J., Gross B., Jacobsen A., Reva B., Schultz N., Onur Sumer S., Sun Y., Chan T. A., Morris L. G., Stuart J., Benz S., Ng S., Benz C., Yau C., Baylin S. B., Cope L., Danilova L., Herman J. G., Bootwalla M., Maglinte D. T., Laird P. W., Triche T., Weisenberger D. J., Van Den Berg D. J., Agrawal N., Bishop J., Boutros P. C., Bruce J. P., Averett Byers L., Califano J., Carey T. E., Chen Z., Cheng H., Chiosea S. I., Cohen E., Diergaarde B., Marie Egloff A., El-Naggar A. K., Ferris R. L., Frederick M. J., Grandis J. R., Guo Y., Haddad R. I., Hammerman P. S., Harris T., Neil Hayes D., Hui A. B. Y., Jack Lee J., Lippman S. M., Liu F.-F., McHugh J. B., Myers J., Kwok Shing Ng P., Perez-Ordonez B., Pickering C. R., Prystowsky M., Romkes M., Saleh A. D., Sartor M. A., Seethala R., Seiwert T. Y., Si H., Tward A. D., Van Waes C., Waggott D. M., Wiznerowicz M., Yarbrough W. G., Zhang J., Zuo Z., Burnett K., Crain D., Gardner J., Lau K., Mallery D., Morris S., Paulauskis J., Penny R., Shelton C., Shelton T., Sherman M., Yena P., Black A. D., Bowen J., Frick J., Gastier-Foster J. M., Harper H. A., Leraas K., Lichtenberg T. M., Ramirez N. C., Wise L., Zmuda E., Baboud J., Jensen M. A., Kahn A. B., Pihl T. D., Pot D. A., Srinivasan D., Walton J. S., Wan Y., Burton R. A., Davidsen T., Demchok J. A., Eley G., Ferguson M. L., Mills Shaw K. R., Ozenberger B. A., Sheth M., Sofia H. J., Tarnuzzer R., Wang Z., Yang L., Claude Zenklusen J., Saller C., Tarvin K., Chen C., Bollag R., Weinberger P., Golusiński W., Golusiński P., Ibbs M., Korski K., Mackiewicz A., Suchorska W., Szybiak B., Wiznerowicz M., Burnett K., N. The Cancer Genome Atlas, I. Genome sequencing centre: Broad, c. Genome, B. C. C. A. data analysis centres, S. B. Harvard Medical, M. D. A. C. C. Women’s Hospital, M. D. A. C. C. The University of Texas, K. University of, H. University of North Carolina at Chapel, I. Broad, M. Baylor College of, C. Memorial Sloan-Kettering Cancer, I. University of California Santa Cruz/Buck, C. Johns Hopkins University/Sidney Kimmel Comprehensive Cancer, C. University of Southern, g. Disease working, C. Biospecimen core resource: International Genomics, H. Nationwide Children’s, c. Data coordinating, o. Project, S. Tissue source sites: Analytical Biological, C. Fred Hutchinson Cancer Research, U. Georgia Regents, C. Greater Poland Cancer, C. International Genomics, Comprehensive genomic characterization of head and neck squamous cell carcinomas. Nature 517, 576–582 (2015). 25631445 10.1038/nature14129 PMC4311405

[68] Williams E. D., Gao D., Redfern A., Thompson E. W., Controversies around epithelial–mesenchymal plasticity in cancer metastasis. Nature Reviews Cancer 19, 716–732 (2019). 31666716 10.1038/s41568-019-0213-x PMC7055151

[69] Tang S., Tao M., McCoy J. P. Jr., Zheng Z. M., The E7 oncoprotein is translated from spliced E6*I transcripts in high-risk human papillomavirus type 16- or type 18-positive cervical cancer cell lines via translation reinitiation. J Virol 80, 4249–4263 (2006). 16611884 10.1128/JVI.80.9.4249-4263.2006 PMC1472016

[70] Ajiro M., Jia R., Zhang L., Liu X., Zheng Z.-M., Intron Definition and a Branch Site Adenosine at nt 385 Control RNA Splicing of HPV16 E6*I and E7 Expression. PLOS ONE 7, e46412 (2012). 23056301 10.1371/journal.pone.0046412 PMC3464268

[71] Stacey Simon N., Jordan D., Williamson Andrew J. K., Brown M., Coote Joanna H., Arrand John R., Leaky Scanning Is the Predominant Mechanism for Translation of Human Papillomavirus Type 16 E7 Oncoprotein from E6/E7 Bicistronic mRNA. Journal of Virology 74, 7284–7297 (2000). 10906182 10.1128/jvi.74.16.7284-7297.2000 PMC112249

[72] del Moral-Hernández O., López-Urrutia E., Bonilla-Moreno R., Martínez-Salazar M., Arechaga-Ocampo E., Berumen J., Villegas-Sepúlveda N., The HPV-16 E7 oncoprotein is expressed mainly from the unspliced E6/E7 transcript in cervical carcinoma C33-A cells. Archives of Virology 155, 1959–1970 (2010). 20865289 10.1007/s00705-010-0787-9

[73] Havens M. A., Hastings M. L., Splice-switching antisense oligonucleotides as therapeutic drugs. Nucleic Acids Research 44, 6549–6563 (2016). 27288447 10.1093/nar/gkw533 PMC5001604

[74] Corallino S., Malabarba M. G., Zobel M., Di Fiore P. P., Scita G., Epithelial-to-Mesenchymal Plasticity Harnesses Endocytic Circuitries. Frontiers in Oncology **Volume** 5 - 2015, (2015). 10.3389/fonc.2015.00045 PMC4341543 25767773

[75] Paterson A. D., Parton R. G., Ferguson C., Stow J. L., Yap A. S., Characterization of E-cadherin Endocytosis in Isolated MCF-7 and Chinese Hamster Ovary Cells: THE INITIAL FATE OF UNBOUND E-CADHERIN *. Journal of Biological Chemistry 278, 21050–21057 (2003). 12657640 10.1074/jbc.M300082200

[76] Song S., Eckerle S., Onichtchouk D., Marrs James A., Nitschke R, Driever W, Pou5f1-Dependent EGF Expression Controls E-Cadherin Endocytosis, Cell Adhesion, and Zebrafish Epiboly Movements. Developmental Cell 24, 486–501 (2013). 23484854 10.1016/j.devcel.2013.01.016 PMC3598594

[77] McCluskey A., Daniel J. A., Hadzic G., Chau N., Clayton E. L., Mariana A., Whiting A., Gorgani N. N., Lloyd J., Quan A., Moshkanbaryans L., Krishnan S., Perera S., Chircop M., von Kleist L., McGeachie A. B., Howes M. T., Parton R. G., Campbell M., Sakoff J. A., Wang X., Sun J.-Y., Robertson M. J., Deane F. M., Nguyen T. H., Meunier F. A., Cousin M. A., Robinson P. J., Building a Better Dynasore: The Dyngo Compounds Potently Inhibit Dynamin and Endocytosis. Traffic 14, 1272–1289 (2013). 24025110 10.1111/tra.12119 PMC4138991

[78] Chen H. R., Yeh Y. C., Liu C. Y., Wu Y. T., Lo F. Y., Tang M. J., Wang Y. K., DDR1 promotes E-cadherin stability via inhibition of integrin-β1-Src activation-mediated E-cadherin endocytosis. Sci Rep 6, 36336 (2016). 27824116 10.1038/srep36336 PMC5099905

[79] Koneva L. A., Zhang Y., Virani S., Hall P. B., McHugh J. B., Chepeha D. B., Wolf G. T., Carey T. E., Rozek L. S., Sartor M. A., HPV Integration in HNSCC Correlates with Survival Outcomes, Immune Response Signatures, and Candidate Drivers. Molecular Cancer Research 16, 90–102 (2018). 28928286 10.1158/1541-7786.MCR-17-0153 PMC5752568

[80] Labarge B., Hennessy M., Zhang L., Goldrich D., Chartrand S., Purnell C., Wright S., Goldenberg D., Broach J. R., Human Papillomavirus Integration Strictly Correlates with Global Genome Instability in Head and Neck Cancer. Molecular Cancer Research 20, 1420–1428 (2022). 35657601 10.1158/1541-7786.MCR-21-0831 PMC9437566

[81] Nulton T. J., Kim N.-K., DiNardo L. J., Morgan I. M., Windle B., Patients with integrated HPV16 in head and neck cancer show poor survival. Oral Oncology 80, 52–55 (2018). 29706188 10.1016/j.oraloncology.2018.03.015 PMC5930384

[82] Friedl P., Alexander S., Cancer Invasion and the Microenvironment: Plasticity and Reciprocity. Cell 147, 992–1009 (2011). 22118458 10.1016/j.cell.2011.11.016

[83] Ribatti D., Tamma R., Annese T., Epithelial-Mesenchymal Transition in Cancer: A Historical Overview. Translational Oncology 13, 100773 (2020). 32334405 10.1016/j.tranon.2020.100773 PMC7182759

[84] Kisoda S., Shao W., Fujiwara N., Mouri Y., Tsunematsu T., Jin S., Arakaki R., Ishimaru N., Kudo Y., Prognostic value of partial EMT-related genes in head and neck squamous cell carcinoma by a bioinformatic analysis. Oral Diseases 26, 1149–1156 (2020). 32277532 10.1111/odi.13351

[85] Williams V. M., Filippova M., Filippov V., Payne K. J., Duerksen-Hughes P., Human Papillomavirus Type 16 E6* Induces Oxidative Stress and DNA Damage. Journal of Virology 88, 6751–6761 (2014). 24696478 10.1128/JVI.03355-13 PMC4054338

[86] Harbison R. A., Kubik M., Konnick E. Q., Zhang Q., Lee S.-G., Park H., Zhang J., Carlson C. S., Chen C., Schwartz S. M., Rodriguez C. P., Duvvuri U., Méndez E., The mutational landscape of recurrent versus nonrecurrent human papillomavirus–related oropharyngeal cancer. JCI Insight 3, (2018). 10.1172/jci.insight.99327 PMC6124437 30046007

[87] Kim M. H., Kim J.-H., Lee J. M., Choi J. W., Jung D., Cho H., Kang H., Hong M. H., Heo S. J., Kim S. H., Choi E. C., Kim D. H., Park Y. M., Kim S., Yoon S. O., Koh Y. W., Cho B. C., Kim H. R., Molecular subtypes of oropharyngeal cancer show distinct immune microenvironment related with immune checkpoint blockade response. British Journal of Cancer 122, 1649–1660 (2020). 32235905 10.1038/s41416-020-0796-8 PMC7251088

[88] Zeng P. Y. F., Cecchini M. J., Barrett J. W., Shammas-Toma M., De Cecco L., Serafini M. S., Cavalieri S., Licitra L., Hoebers F., Brakenhoff R. H., Leemans C. R., Scheckenbach K., Poli T., Wang X., Liu X., Laxague F., Prisman E., Poh C., Bose P., Dort J. C., Shaikh M. H., Ryan S. E. B., Dawson A., Khan M. I., Howlett C. J., Stecho W., Plantinga P., Daniela da Silva S., Hier M., Khan H., MacNeil D., Mendez A., Yoo J., Fung K., Lang P., Winquist E., Palma D. A., Ziai H., Amelio A. L., Li S. S. C., Boutros P. C., Mymryk J. S., Nichols A. C., Immune-based classification of HPV-associated oropharyngeal cancer with implications for biomarker-driven treatment de-intensification. eBioMedicine 86, (2022). 10.1016/j.ebiom.2022.104373 PMC9706534 36442320

[89] Zhang P., Li S., Zhang T., Cui F., Shi J.-H., Zhao F., Sheng X., Characterization of Molecular Subtypes in Head and Neck Squamous Cell Carcinoma With Distinct Prognosis and Treatment Responsiveness. Frontiers in Cell and Developmental Biology 9, (2021). 10.3389/fcell.2021.711348 PMC8476885 34595167

[90] Mino-Kenudson M., Immunohistochemistry for predictive biomarkers in non-small cell lung cancer. Transl Lung Cancer Res 6, 570–587 (2017). 29114473 10.21037/tlcr.2017.07.06 PMC5653529

[91] Sorokin M., Ignatev K., Poddubskaya E., Vladimirova U., Gaifullin N., Lantsov D., Garazha A., Allina D., Suntsova M., Barbara V., Buzdin A., RNA Sequencing in Comparison to Immunohistochemistry for Measuring Cancer Biomarkers in Breast Cancer and Lung Cancer Specimens. Biomedicines 8, (2020). 10.3390/biomedicines8050114 PMC7277916 32397474

[92] Aartsma-Rus A., van Vliet L., Hirschi M., Janson A. A. M., Heemskerk H., de Winter C. L., de Kimpe S., van Deutekom J. C. T., t Hoen P. A. C., van Ommen G.-J. B., Guidelines for Antisense Oligonucleotide Design and Insight Into Splice-modulating Mechanisms. Molecular Therapy 17, 548–553 (2009). 18813282 10.1038/mt.2008.205 PMC2835096

[93] Cerasuolo A., Annunziata C., Tortora M., Starita N., Stellato G., Greggi S., Maglione M. G., Ionna F., Losito S., Botti G., Buonaguro L., Buonaguro F. M., Tornesello M. L., Comparative analysis of HPV16 gene expression profiles in cervical and in oropharyngeal squamous cell carcinoma. Oncotarget 8, (2017). 10.18632/oncotarget.15977 PMC5470952 28423662

[94] Mahal B. A., Catalano P. J., Haddad R. I., Hanna G. J., Kass J. I., Schoenfeld J. D., Tishler R. B., Margalit D. N., Incidence and Demographic Burden of HPV-Associated Oropharyngeal Head and Neck Cancers in the United States. Cancer Epidemiology, Biomarkers & Prevention 28, 1660–1667 (2019). 10.1158/1055-9965.EPI-19-0038 31358520

[95] Van Tubergen E., Vander Broek R., Lee J., Wolf G., Carey T., Bradford C., Prince M., Kirkwood K. L., D’Silva N. J., Tristetraprolin regulates interleukin-6, which is correlated with tumor progression in patients with head and neck squamous cell carcinoma. Cancer 117, 2677–2689 (2011). 21656745 10.1002/cncr.25859 PMC3574798

[96] Brenner J. C., Graham M. P., Kumar B., Saunders L. M., Kupfer R., Lyons R. H., Bradford C. R., Carey T. E., Genotyping of 73 UM-SCC head and neck squamous cell carcinoma cell lines. Head & Neck 32, 417–426 (2010). 19760794 10.1002/hed.21198 PMC3292176

[97] Tang A. L., Hauff S. J., Owen J. H., Graham M. P., Czerwinski M. J., Park J. J., Walline H., Papagerakis S., Stoerker J., McHugh J. B., Chepeha D. B., Bradford C. R., Carey T. E., Prince M. E., UM-SCC-104: A New human papillomavirus-16–positive cancer stem cell–containing head and neck squamous cell carcinoma cell line. Head & Neck 34, 1480–1491 (2012). 22162267 10.1002/hed.21962 PMC3369005

[98] Symer D. E., Akagi K., Geiger H. M., Song Y., Li G., Emde A.-K., Xiao W., Jiang B., Corvelo A., Toussaint N. C., Li J., Agrawal A., Ozer E., El-Naggar A. K., Du Z., Shewale J. B., Stache-Crain B., Zucker M., Robine N., Coombes K. R., Gillison M. L., Diverse tumorigenic consequences of human papillomavirus integration in primary oropharyngeal cancers. Genome Research 32, 55–70 (2022). 34903527 10.1101/gr.275911.121 PMC8744672

[99] Li S., Garb B. F., Qin T., Soppe S., Lopez E., Patil S., D’Silva N. J., Rozek L. S., Sartor M. A., Tumor Subtype Classification Tool for HPV-associated Head and Neck Cancers. bioRxiv, 2024.2007.2005.601906 (2024). 10.1016/j.oraloncology.2025.107726 PMC13274521 41045845

[100] Barik S., Site-directed mutagenesis in vitro by megaprimer PCR. Methods Mol Biol 57, 203–215 (1996). 8850007 10.1385/0-89603-332-5:203

[101] Au - Partridge J., Au - Flaherty P., An In vitro FluoroBlok Tumor Invasion Assay. JoVE, e1475 (2009). 10.3791/1475 PMC3148933 19620957

[102] Schmitd L. B., Beesley L. J., Russo N., Bellile E. L., Inglehart R. C., Liu M., Romanowicz G., Wolf G. T., Taylor J. M. G., D’Silva N. J., Redefining Perineural Invasion: Integration of Biology With Clinical Outcome. Neoplasia 20, 657–667 (2018). 29800815 10.1016/j.neo.2018.04.005 PMC6030236

[103] Bankhead P., Loughrey M. B., Fernández J. A., Dombrowski Y., McArt D. G., Dunne P. D., McQuaid S., Gray R. T., Murray L. J., Coleman H. G., James J. A., Salto-Tellez M., Hamilton P. W., QuPath: Open source software for digital pathology image analysis. Scientific Reports 7, 16878 (2017). 29203879 10.1038/s41598-017-17204-5 PMC5715110

[104] S. A. (2010).

[105] Martin M., Cutadapt removes adapter sequences from high-throughput sequencing reads. 2011 17, 3 (2011).

[106] Zhang Y., Parmigiani G., Johnson W. E., ComBat-seq: batch effect adjustment for RNA-seq count data. NAR Genomics and Bioinformatics 2, (2020). 10.1093/nargab/lqaa078 PMC7518324 33015620

[107] Robinson M. D., McCarthy D. J., Smyth G. K., edgeR: a Bioconductor package for differential expression analysis of digital gene expression data. Bioinformatics 26, 139–140 (2009). 19910308 10.1093/bioinformatics/btp616 PMC2796818

[108] McCarthy D. J., Chen Y., Smyth G. K., Differential expression analysis of multifactor RNA-Seq experiments with respect to biological variation. Nucleic Acids Research 40, 4288–4297 (2012). 22287627 10.1093/nar/gks042 PMC3378882

[109] Chen Y., Lun A. T., Smyth G. K., From reads to genes to pathways: differential expression analysis of RNA-Seq experiments using Rsubread and the edgeR quasi-likelihood pipeline. F1000Res 5, 1438 (2016). 27508061 10.12688/f1000research.8987.1 PMC4934518

[110] Li H., Handsaker B., Wysoker A., Fennell T., Ruan J., Homer N., Marth G., Abecasis G., Durbin R., The Sequence Alignment/Map format and SAMtools. Bioinformatics 25, 2078–2079 (2009). 19505943 10.1093/bioinformatics/btp352 PMC2723002

[111] Danecek P., Bonfield J. K., Liddle J., Marshall J., Ohan V., Pollard M. O., Whitwham A., Keane T., McCarthy S. A., Davies R. M., Li H., Twelve years of SAMtools and BCFtools. GigaScience 10, (2021). 10.1093/gigascience/giab008 PMC7931819 33590861

[112] Rajaby R., Zhou Y., Meng Y., Zeng X., Li G., Wu P., Sung W.-K., SurVirus: a repeat-aware virus integration caller. Nucleic Acids Research 49, e33–e33 (2021). 33444454 10.1093/nar/gkaa1237 PMC8034624

[113] Newman A. M., Steen C. B., Liu C. L., Gentles A. J., Chaudhuri A. A., Scherer F., Khodadoust M. S., Esfahani M. S., Luca B. A., Steiner D., Diehn M., Alizadeh A. A., Determining cell type abundance and expression from bulk tissues with digital cytometry. Nature Biotechnology 37, 773–782 (2019). 10.1038/s41587-019-0114-2 PMC6610714 31061481

[114] Shannon P., Markiel A., Ozier O., Baliga N. S., Wang J. T., Ramage D., Amin N., Schwikowski B., Ideker T., Cytoscape: A Software Environment for Integrated Models of Biomolecular Interaction Networks. Genome Research 13, 2498–2504 (2003). 14597658 10.1101/gr.1239303 PMC403769

[115] Otasek D., Morris J. H., Bouças J., Pico A. R., Demchak B., Cytoscape Automation: empowering workflow-based network analysis. Genome Biology 20, 185 (2019). 31477170 10.1186/s13059-019-1758-4 PMC6717989

