# Supplementary material for: Splicing of HPV16 E6 promotes aggressive invasion in oropharyngeal cancer via endocytosis of E-cadherin
Source: bioRxiv. 2025 Oct 16:2025.10.16.682706. Preprint. [Version 1] doi: 10.1101/2025.10.16.682706 (PMC12632831; doi:10.1101/2025.10.16.682706)
Supplement: Supplement 1 [file media-1.pdf]

Supplementary figures- page 2-19

Supplementary tables- page 19-25

Figure S1

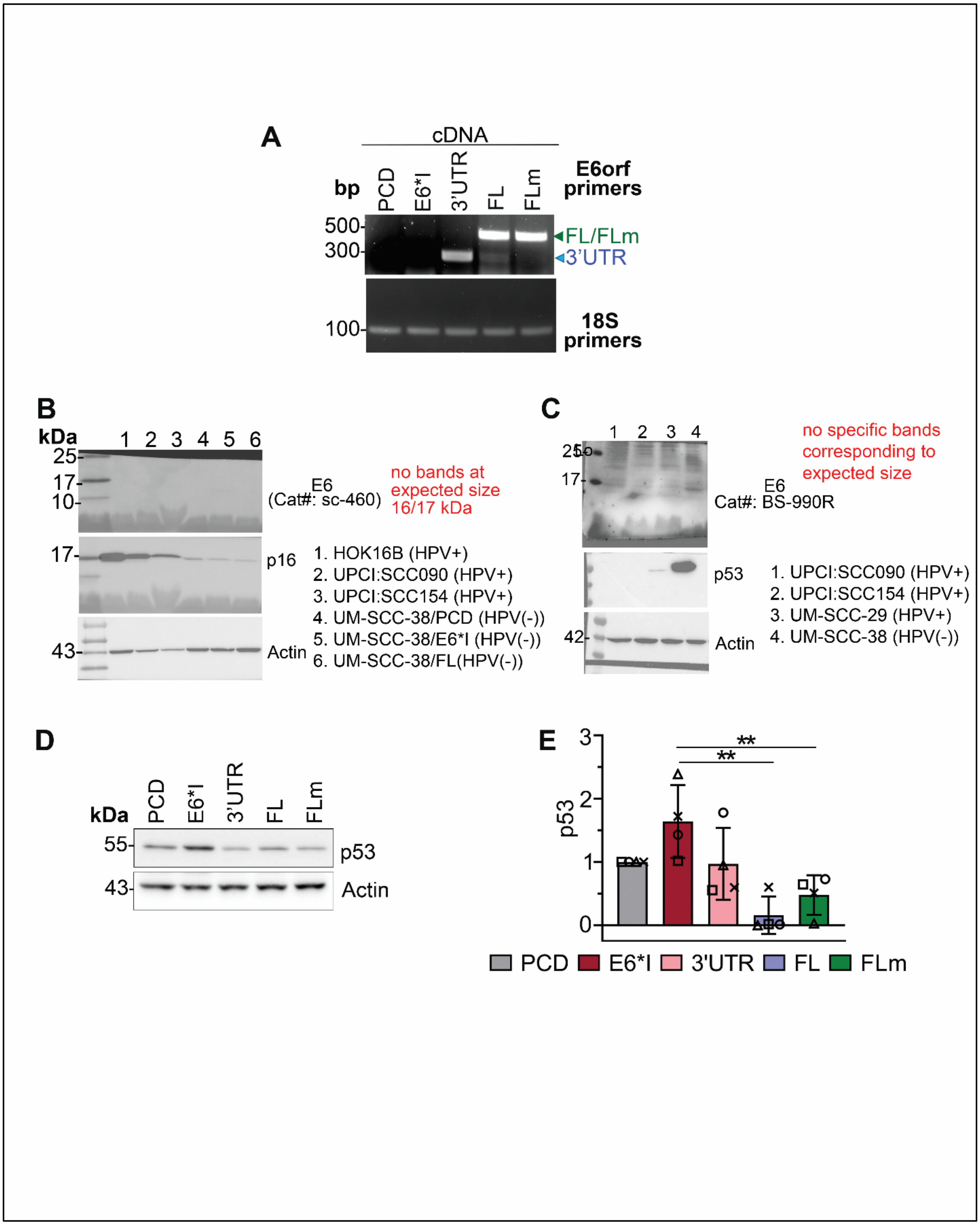
A
cDNA
bp
PCD
E6*I
3'UTR
FL
FLm
E6orf primers
500-
300-
FL/FLm
3'UTR
100-
18S primers
B
kDa
1 2 3 4 5 6
25-
17-
10-
E6 (Cat#: sc-460)
no bands at expected size 16/17 kDa
17-
p16
43-
Actin
1. HOK16B (HPV+)
2. UPCI:SCC090 (HPV+)
3. UPCI:SCC154 (HPV+)
4. UM-SCC-38/PCD (HPV(-))
5. UM-SCC-38/E6*I (HPV(-))
6. UM-SCC-38/FL(HPV(-))
C
1 2 3 4
no specific bands corresponding to expected size
25
17-
E6 Cat#: BS-990R
p53
42-
Actin
1. UPCI:SCC090 (HPV+)
2. UPCI:SCC154 (HPV+)
3. UM-SCC-29 (HPV+)
4. UM-SCC-38 (HPV(-))
D
kDa
PCD
E6*I
3'UTR
FL
FLm
55-
p53
43-
Actin
E
p53
3
2
1
0
**
**
PCD
E6*I
3'UTR
FL
FLm

Fig. S1: Overexpression of HPV16 E6 isoforms in HPV-negative OPSCC cell line, UM-SCC-38.

(A) Representative agarose gel from 3 independent experiments showing transcripts of E6 isoforms using primers against E6 open reading frame (E6orf). Expected size: FL or FLm=477 bp; 3'UTR=295 bp.

(B&C) Raw immunoblots for two commercial E6 antibodies, Santa Cruz (sc-460; B) and Bioss (BS-990R; C). Actin was used as a loading control, p16 confirms presence of HPV in lysates.

(D&E) Immunoblot for p53, the downstream target of E6, in lysates (D). Actin was used as a loading control. Densitometry for p53 (E) was normalized to actin and expressed relative to PCD control. Each shape represents an independent experiment (four). Data are represented as mean ± SD. **$p<0.01$ (One-way ANOVA with post-hoc Tukey test).

Figure S2

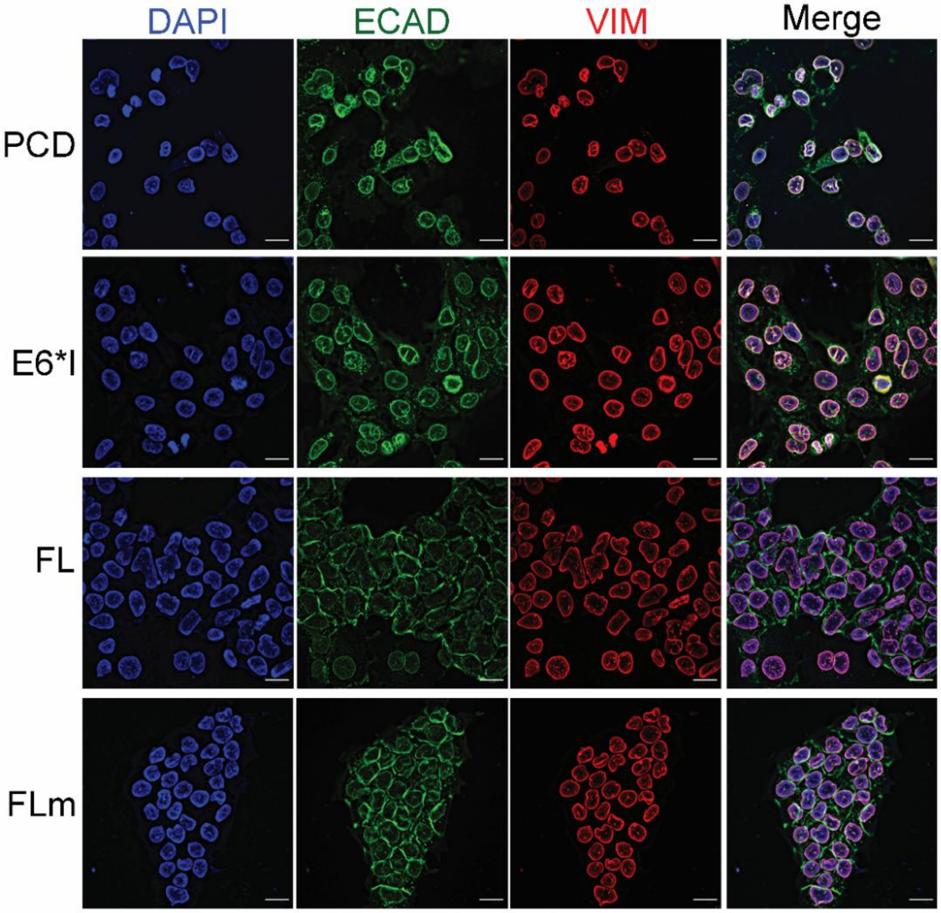

Fig. S2: Individual channels of Immunofluorescence of DAPI (blue), E cadherin (ECAD, green) and vimentin (VIM, red) in UM-SCC-38 stably overexpressing E6 isoforms. Scale bar =100uM

Figure S3

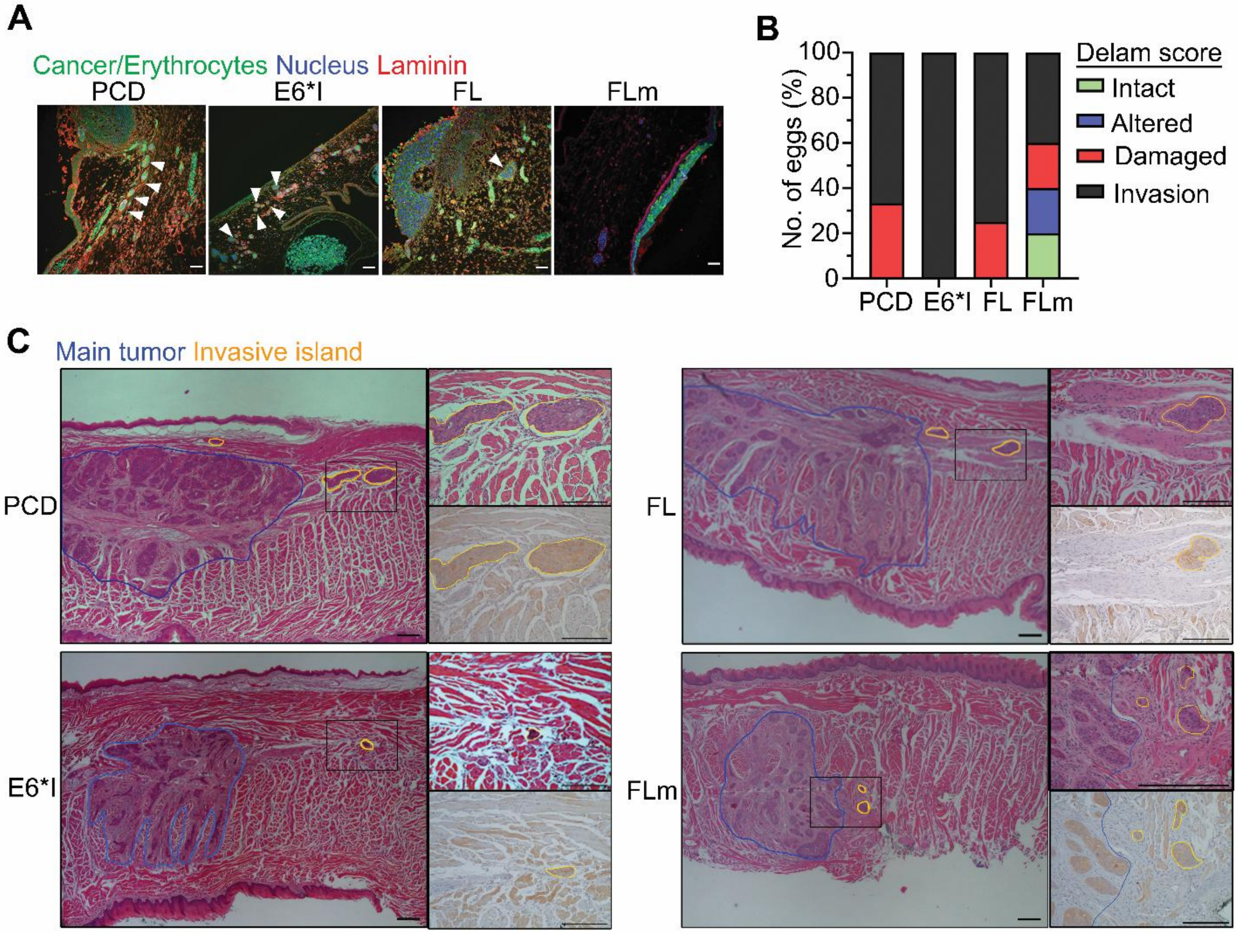
A
Cancer/Erythrocytes Nucleus Laminin
PCD
E6*I
FL
FLm
B
No. of eggs (%)
100
80
60
40
20
0
PCD E6*I FL FLm
Delam score
Intact
Altered
Damaged
Invasion
C
Main tumor Invasive island
PCD
FL
E6*I
FLm

Fig. S3: In vivo CAM and mouse experiments. (A) Representative images of upper CAM stained for laminin (red; basement membrane) and DAPI (blue; nucleus). Green signals highlights fluorescently-labelled tumor cells with some non-specific staining observed in erythrocytes. Arrowheads indicate invasive tumor islands. Scale bar =50μm. (B) Basement membrane degradation of immunofluorescence images (A) was scored according to the CAM-Delam scoring system reported by Palaniappan et al. (2020) (*64*) (Fisher's Exact Score; p=ns). (C) H&E and CK-stained images of main tumor bulk (blue) and invasive islands (yellow) in mouse tongues. Right panel is the enlarged H&E and CK-stained images of boxed area in left panel. Scale bar =200μm.

Figure S4

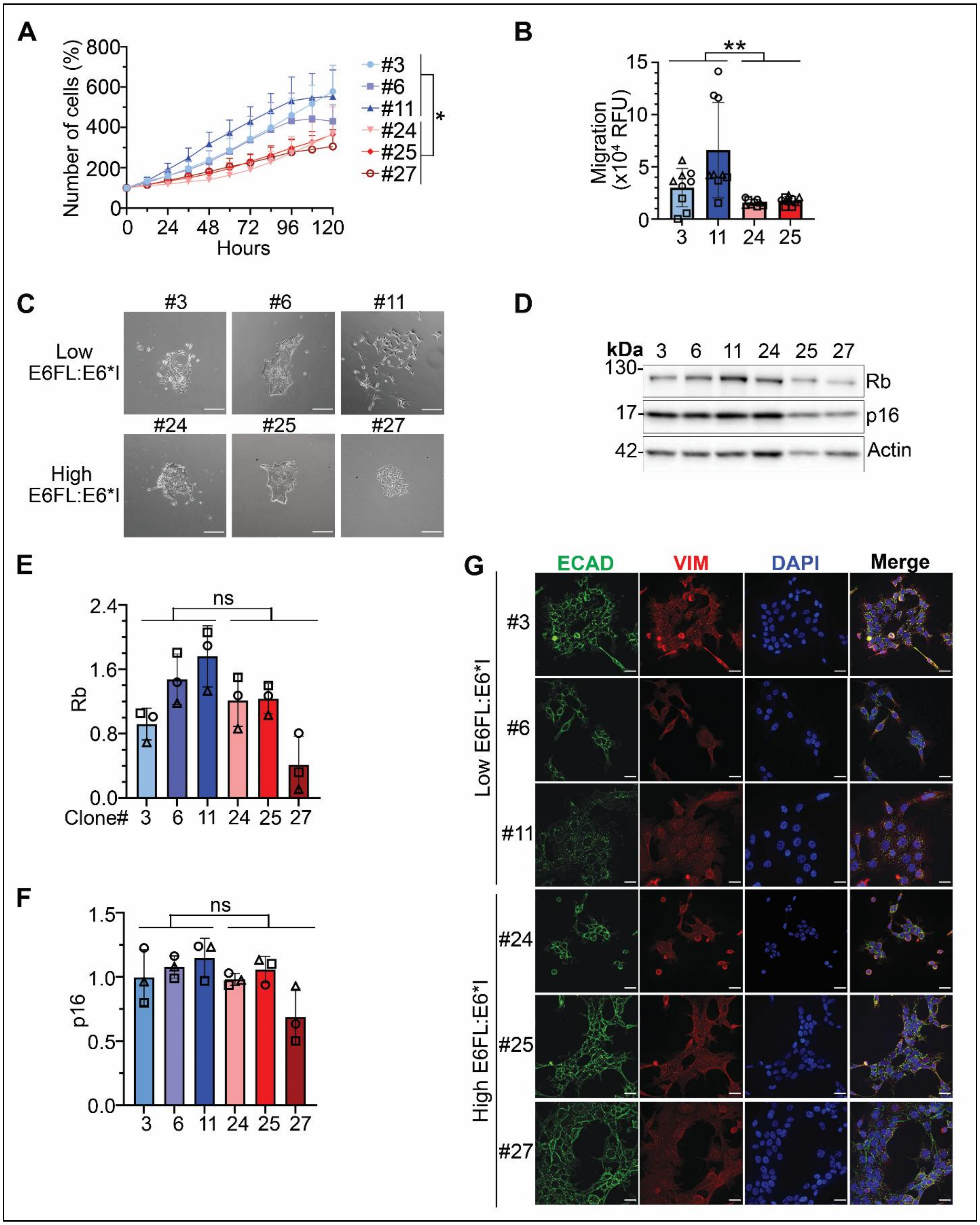
A
Number of cells (%)
Hours
#3
#6
#11
#24
#25
#27
*
B
Migration (x10^4 RFU)
**
3 11 24 25
C
#3 #6 #11
Low E6FL:E6*I
#24 #25 #27
High E6FL:E6*I
D
kDa 3 6 11 24 25 27
130-
Rb
17-
p16
42-
Actin
E
ns
Rb
Clone# 3 6 11 24 25 27
F
ns
p16
3 6 11 24 25 27
G
ECAD
VIM
DAPI
Merge
Low E6FL:E6*I
#3
#6
#11
High E6FL:E6*I
#24
#25
#27

Fig. S4: Validation and functional phenotypes of UPCI:SCC154 single-cell clones

(A) Proliferation assay performed on UPCI:SCC154 single-cell clones. Viable cells were quantified and expressed as percent (%) of 0h.

(B) Fluoroblok transwell migration assay was performed. Migration was calculated by normalizing with relative fluorescence unit (RFU) at 0h. Each shape is an independent experiment (n=3), with three replicates per experiment.

(C) Phase contrast images of colonies after 15d growth. Scale bar = 500μm

(D) Representative immunoblot showing Rb and p16 expression from three independent experiments. Actin was used as the loading control

(E&F) Densitometric quantification of Rb (D) and p16 (E), normalized to actin. Each shape shows one experiment (three). Ns= not significant (unpaired t test)

(G) Individual channels for immunofluorescence staining of DAPI (blue), E cadherin (ECAD, green) and vimentin (VIM, red) in UPCI:SCC154 single-cell clones.

Figure S5

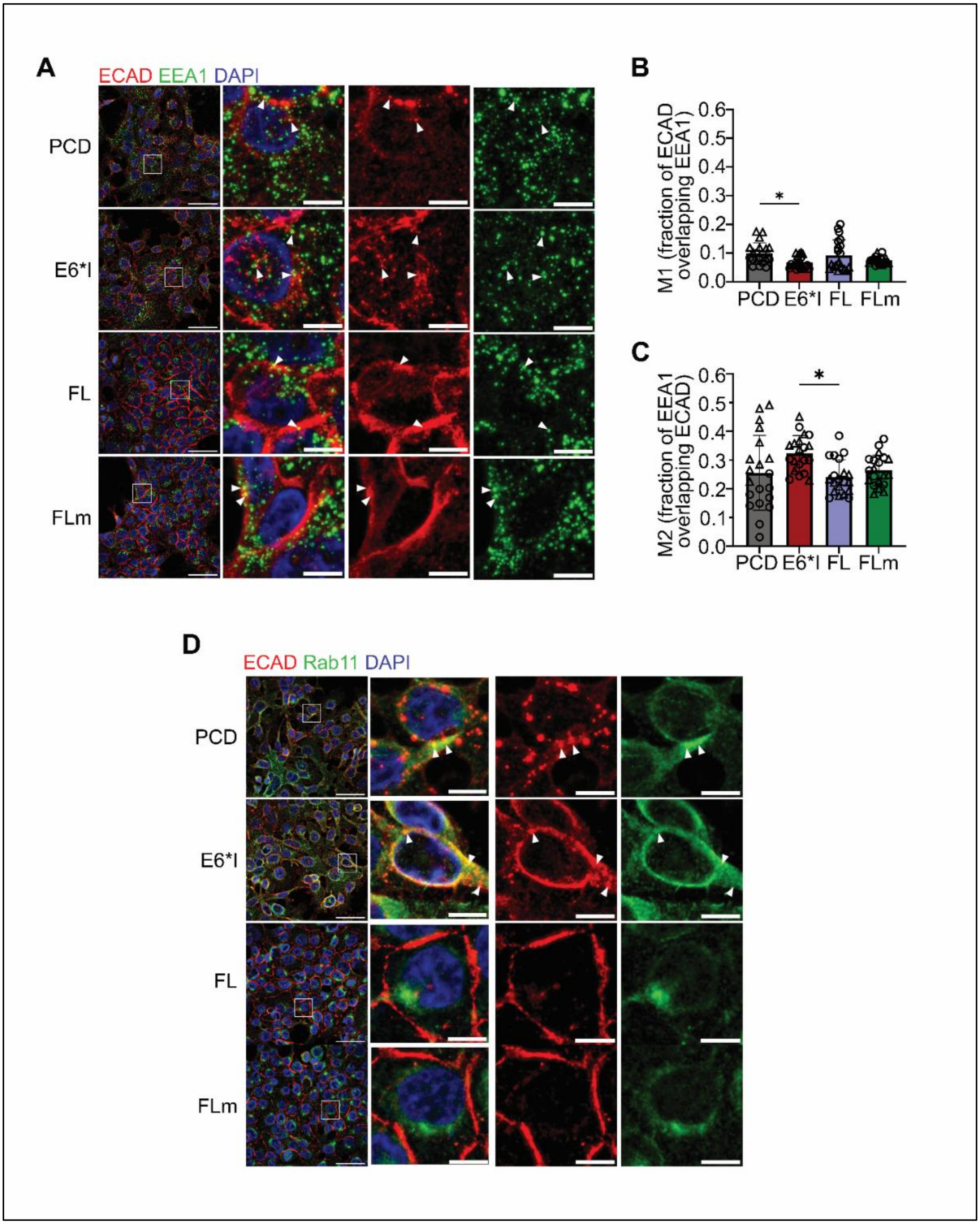
A
ECAD EEA1 DAPI
PCD
E6*I
FL
FLm
B
M1 (fraction of ECAD overlapping EEA1)
0.6
0.5
0.4
0.3
0.2
0.1
0.0
*
PCD E6*I FL FLm
C
M2 (fraction of EEA1 overlapping ECAD)
0.6
0.5
0.4
0.3
0.2
0.1
0.0
*
PCD E6*I FL FLm
D
ECAD Rab11 DAPI
PCD
E6*I
FL
FLm

Fig. S5: Co-localization of ECAD with markers for endocytotic vesicles, EEA1 and Rab11. (A) Maximum projection images of ECAD (red) and EEA1 (green) in UM-SCC-38 expressing E6 isoforms or vector control (PCD). Second panel shows merged image of boxed area in first panel. Third and fourth panels show individual channels for ECAD (red) and EEA1 (green), respectively. Arrowheads show co-localization of ECAD and EEA1. (B&C) Co-localization of ECAD and EEA1 was quantified from 12 z slices, 5 fields/duplicate. Manders' coefficients, i.e. fraction of ECAD overlapping EEA1 (B) and fraction of EEA1 overlapping ECAD (C), were quantified using ImageJ JaCoP plugin. (D) Maximum projection images of ECAD (red) and EEA1 (green) in UM-SCC-38 with PCD or E6 isoforms. Second panel shows enlarged image of boxed area in first panel. Third and fourth panels are ECAD (red) and Rab11 (green), respectively. DAPI (blue) = nucleus. Scale bar = 50μm (left) and 10μm (right).

Figure S6

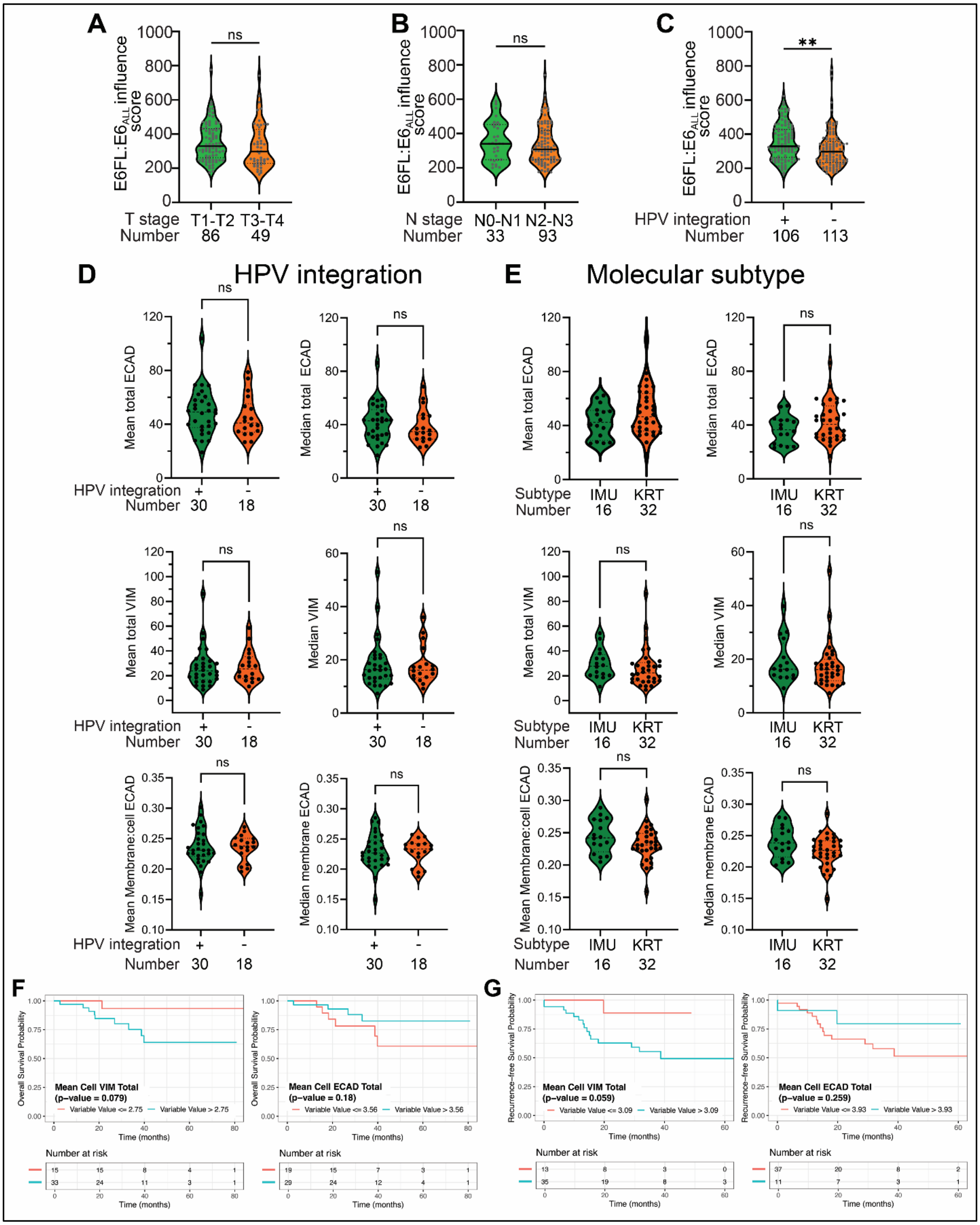

A
B
C
E6FL:E6ALL influence score
ns
**
T stage T1-T2 T3-T4
Number 86 49
N stage N0-N1 N2-N3
Number 33 93
HPV integration + -
Number 106 113
D HPV integration
E Molecular subtype
Mean total ECAD
Median total ECAD
Mean total VIM
Median VIM
Mean Membrane:cell ECAD
Median membrane ECAD
HPV integration + -
Number 30 18
Subtype IMU KRT
Number 16 32
F
G
Overall Survival Probability
Recurrence-free Survival Probability
Mean Cell VIM Total
(p-value = 0.079)
Mean Cell ECAD Total
(p-value = 0.18)
Mean Cell VIM Total
(p-value = 0.059)
Mean Cell ECAD Total
(p-value = 0.259)
Time (months)
Number at risk

Fig. S6: Association of molecular subtype with clinical and histopathologic variables in patients. (A-C) Violin plots showing E6FL:$E6_{ALL}$ influence scores in patients segregated by tumor stage (A), nodal stage (B) and HPV integration status (C). (D-E) Violin plots showing mean and median ECAD, VIM and membrane:cell ECAD in patients segregated by HPV integration status (D) and molecular subtype (E). (F-G) Overall (F) and recurrence-free (G) survival showing segregation of patients with low and high mean cell VIM (left) and ECAD (right). Log-rank test was performed to determine statistical significance.

Figure S7

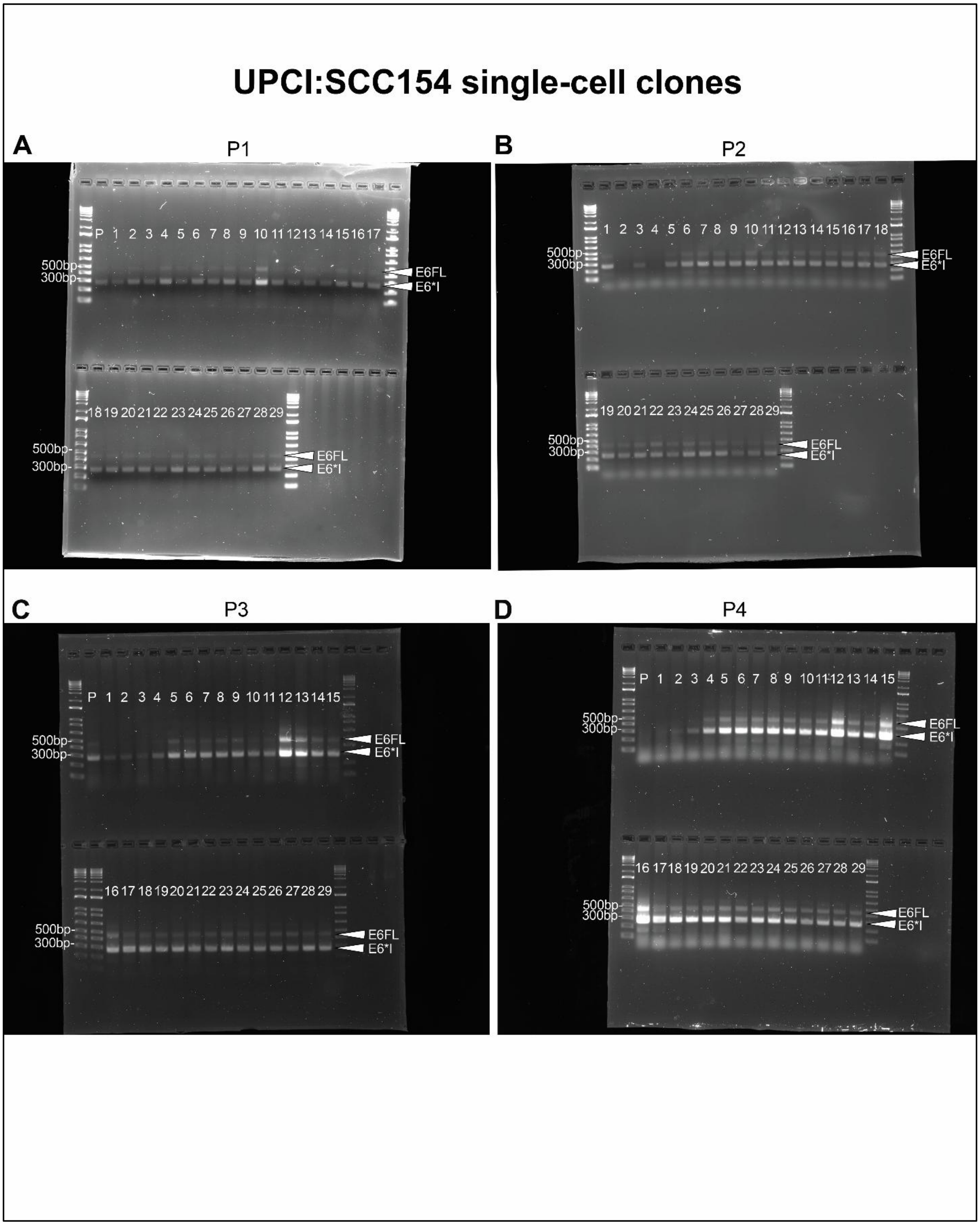
UPCI:SCC154 single-cell clones
A
P1
P 1 2 3 4 5 6 7 8 9 10 11 12 13 14 15 16 17
500bp-
300bp-
E6FL
E6*I
18 19 20 21 22 23 24 25 26 27 28 29
B
P2
1 2 3 4 5 6 7 8 9 10 11 12 13 14 15 16 17 18
19 20 21 22 23 24 25 26 27 28 29
C
P3
P 1 2 3 4 5 6 7 8 9 10 11 12 13 14 15
16 17 18 19 20 21 22 23 24 25 26 27 28 29
D
P4
P 1 2 3 4 5 6 7 8 9 10 11 12 13 14 15
16 17 18 19 20 21 22 23 24 25 26 27 28 29

Fig. S7: Raw data for screening of UPCI:SCC154SC clones in Fig. 4B.

Single cells from UPCI:SCC154 parent cell line were grown separately. 29 clones were isolated from single cells. RT-PCR was performed using E6orf primers and PCR products were subjected to agarose gel electrophoresis. E6FL: (E6FL+E6*I) ratios were expressed as densitometric units of E6FL divided by the sum of densitometric units of E6FL and E6*I. Four consecutive passages (P1 - P4) were screened. P= UPCI:SCC154 parent cell line

Figure S8

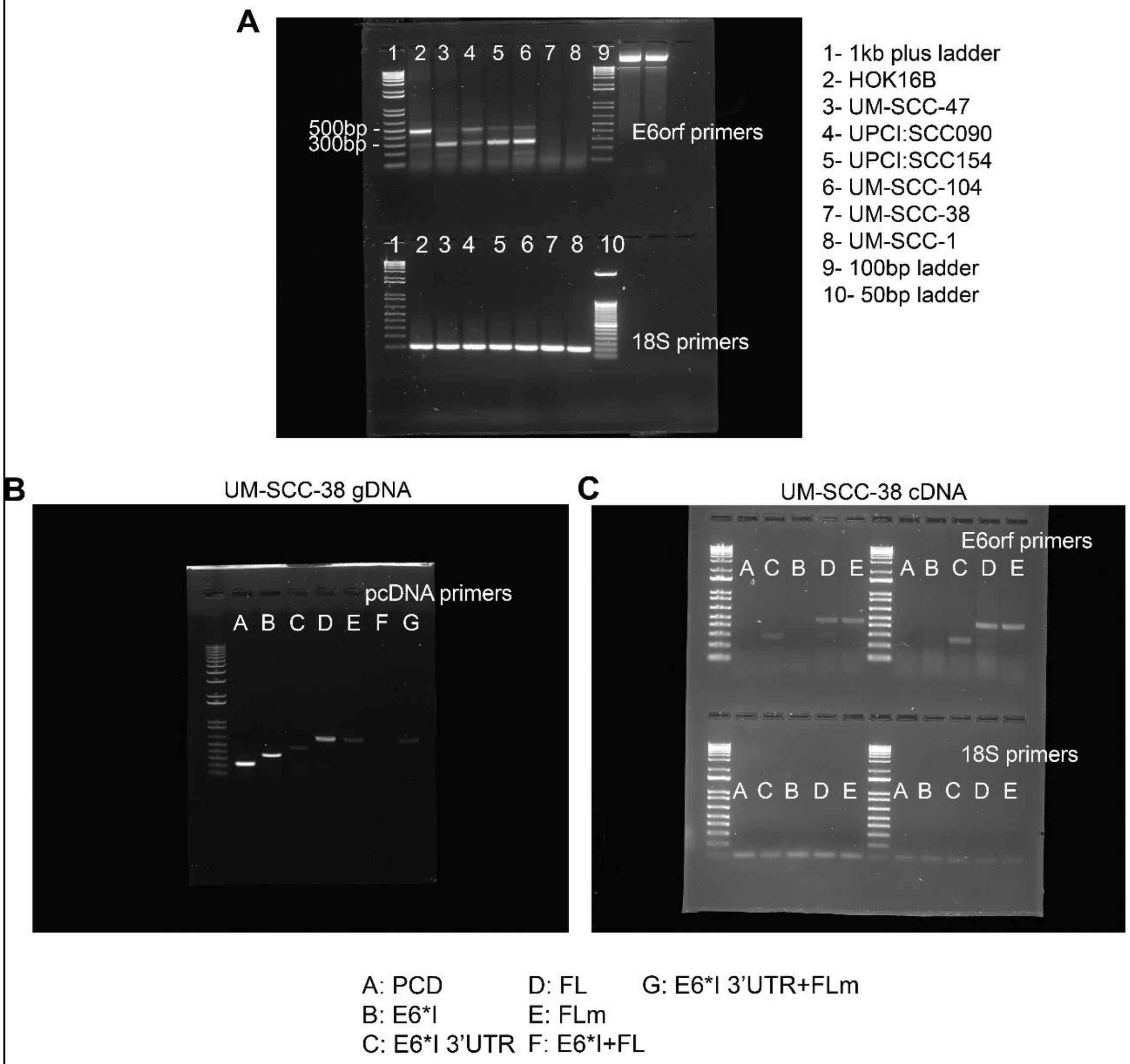

Fig. S8: Uncropped images of agarose gel for RT-PCR experiments. (A) Uncropped image for Fig. 1B. (B) Uncropped image for Fig. 2B. (C) Uncropped image for Fig. S1A

Figure S9

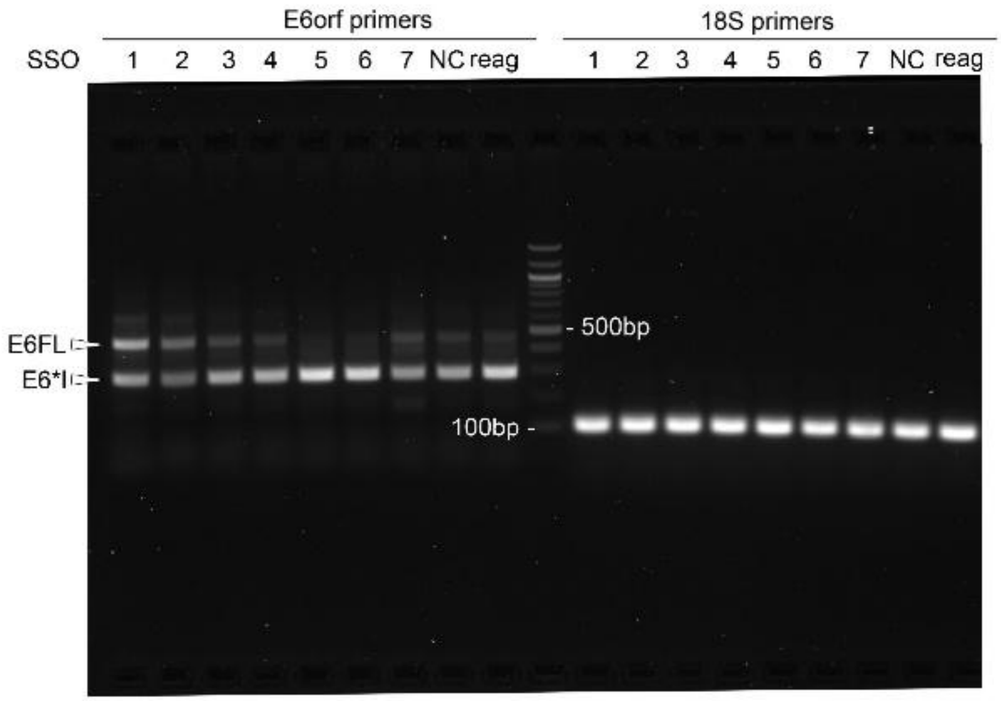

Fig. S9: Uncropped image of agarose gel for Fig. 5B.

Figure S10

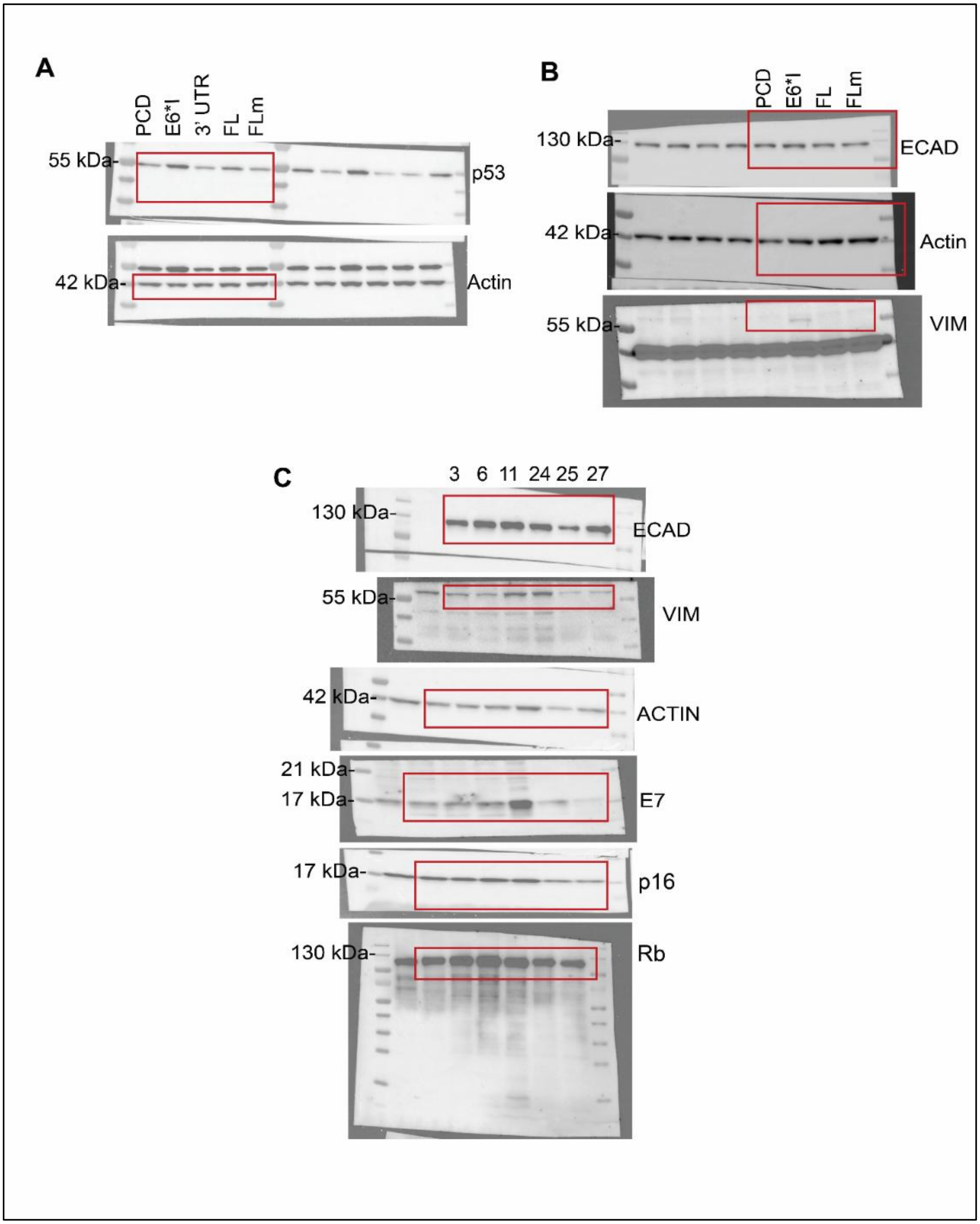

Fig. S10: Raw immunoblot images for (A) Fig. S1D, (B) Fig. 2L and (C) Fig. 4F. The raw

chemiluminescence image was merged with the colorimetric image on ImageLab 6.0.1. Red box represents the bands and lanes used for the main relevant figure.

Supplementary Tables

Table S1: Clinical cohorts and anatomic subsites of patients

	**UM18**	**UM67**	**HVC**	**TCGA**	**Sum**	**Used in Survival**
Hypopharynx	0	0	0	2	2	2
Larynx	0	0	0	1	1	1
Oral Cavity	1	0	0	16	17	17
Oropharynx	17	52	83	47	199	116
**Sum**	18	52	83	66	**219**	**136**
Used in Survival	18	52	0	66	**136**	-

Table S2: Clinical cohorts and HPV genotypes of patients

	**UM18**	**UM67**	**HVC**	**TCGA**	**Sum**	**Used in Survival**
HPV16	14	51	74	55	194	120
HPV18	1	0	2	0	3	1
HPV33	1	1	4	8	14	10
HPV35	2	0	2	3	7	5
HPV59	0	0	1	0	1	0
**Sum**	18	52	83	66	**219**	**136**
Used in Survival	18	52	0	66	**136**	-

Table S3: Statistics for association network in Fig. 7D

Variable 1	Variable 2	estimate	p.value	statistic	std.error
E6FL:E6$_{ALL}$ Influence Score	CD4 Tcells	385.751799	3.64E-05	4.21760962	91.462187
E6FL:E6$_{ALL}$ Influence Score	CD8 Tcells	579.496904	0.0043864	2.87959168	201.242735
Epithelial Mesenchymal Transition	E6FL:E6$_{ALL}$ Influence Score	0.00163075	0.01307213	2.50272067	6.52E-04
Bcell Score	E6FL:E6$_{ALL}$ Influence Score	0.0037441	7.95E-10	6.43537194	5.82E-04
Respiratory Electron Transport Chain	E6FL:E6$_{ALL}$ Influence Score	-0.0043895	5.77E-13	-7.6755958	5.72E-04
E6FL:E6$_{ALL}$ Influence Score	Focal Adhesion	19.0962538	0.00650249	2.74827268	6.94845671
Oxidative Phosphorylation Score	E6FL:E6$_{ALL}$ Influence Score	-0.0046822	3.18E-15	-8.5031948	5.51E-04
Keratinization Score	E6FL:E6$_{ALL}$ Influence Score	-0.0019376	0.00183559	-3.1550409	6.14E-04
E6FL:E6$_{ALL}$ Influence Score	Dendritic Cells	1552.92204	1.11E-07	5.49462007	282.625918
E6FL:E6$_{ALL}$ Influence Score	3 yr Survival	102.435113	9.39E-04	3.46427219	29.569014

Variable 1	Variable 2	estimate	p.value	statistic	std.error
E6FL:$E6_{ALL}$ Influence Score	p-EMT Score	-16.2575	0.02029723	-2.3382462	6.95286058
Subtype	E6FL:$E6_{ALL}$ Influence Score	0.01143914	2.21E-05	4.24227298	0.00269646
Tcell Score	E6FL:$E6_{ALL}$ Influence Score	0.00234137	3.05E-04	3.67087703	6.38E-04
Sex	E6FL:$E6_{ALL}$ Influence Score	-0.7091005	0.00659748	-2.7165071	0.26103391
E6FL:$E6_{ALL}$ Influence Score	Cell Adhesion	21.111515	0.00556508	2.80076294	7.53777289
E6FL:$E6_{ALL}$ Influence Score	E6FL:$E6_{ALL}$ Expression ratio	157.363936	0.00246447	3.06402604	51.3585505
E6FL:$E6_{ALL}$ Influence Score	Fibroblast Cells	329.1033	0.00371462	2.93368192	112.180976
HPV Integration	E6FL:$E6_{ALL}$ Influence Score	-0.0050013	7.68E-04	-3.3641456	0.00148663
E6FL:$E6_{ALL}$ Influence Score	Macrophage Cells	240.27648	0.02245527	2.29925311	104.50197
E6FL:$E6_{ALL}$ Influence Score	Malignant Cells	-138.59057	9.48E-06	-4.5377549	30.5416608

Table S4: Primers for generation of plasmids

Primers	Sequences
E6-BamHI-F	AGATGGATCCTTTTATGCACCAAAAGAGAAC
E6FL-EcoRI-R	TAAGAATTCCTTACAGCTGGGTTTCTCTAC
E6*I-EcoRI-R	TAAGAATTCCGTTAATACACCTCACGTCGCAGTAACTG
E6FLm-BamHI-F	CAAGCTTGGTACCGAGCTCGGATCCTTTTATGCACCAAAAG
E6FLm-EcoRI-R	AGTGTGATGGATATCTGCAGAATTCCTTACAGCTGGGTTTC
E6-splice-mutant-mega-R	AAAGCAAAGTCATATAGCTCGCGTCGCAGTAACTGT
E6*I-3'UTR-BsmGI-F	CAAGCAACAGTTACTGCGACGTGAGGTGTATTAACTGTCAAAAGCCACTGTGTCC
E6*I-3'UTR-EcoRI-R	GAAACCCAGCTGTAAGGAATTCTGCAGATATCCATCACACT

Table S5: SSOs and sequences

SSO name	Sequence
SSO-1	TACCTCACGTCGCAGTAACT
SSO-2	AGTCATATACCTCACGTCGC
SSO-3	TATACTATGCATAAATCCCGAA
SSO-4	TCTCTATATACTATGCATAAAT
SSO-5	AATGTCTATACTCACTAATTTT
SSO-6	TAATGTTGTTCCATACAAACTA
SSO-7	TAATACACCTAATTAACAAATC
Negative control	CCTATAGGACTATCCAGGAA

Table S6: Primers for PCR and qPCR

Primer name	Forward sequence	Reverse sequence
pcDNA	CCCACTGCTTACTGGCTTATC	GCAACTAGAAGGCACAGTCG
E6orf	ATGCACCAAAAGAGAACTGC	TTACAGCTGGGTTTCTCTACGTGT
18S	CTTAGAGGGACAAGTGGCG	ACGCTGAGCCAGTCAGTGTA
E6FL	ACAAACCGTTGTGTGATTTGTT	CAGTGGCTTTTGACAGTTAATACA
E6ALL	ATGCACAGAGCTGCAAACAA	TCACGTCGCAGTAACTGTTG
E6*I	ATGCACCAAAAGAGAACTGC (same as E6orf-F)	TAATACACCTCACGTCGCAG
Actin	AAATCGTGCGTGACATCAAAGA	GCCATCTCCTGCTTCGAAGTC
